# MyGood Trip, a Telemedicine Intervention for Physical Activity Recovery After Bariatric Surgery: Randomized Controlled Trial

**DOI:** 10.2196/26077

**Published:** 2023-03-28

**Authors:** Katia Lurbe i Puerto, Matthieu Bruzzi, Claire Rives-Lange, Tigran Poghosyan, Marion Bretault, Gilles Chatellier, Aurelie Vilfaillot, Jean-Marc Chevallier, Sebastien Czernichow, Claire Carette

**Affiliations:** 1 Service de Nutrition, Centre Spécialisé Obésité Hôpital Européen Georges Pompidou Assistance Publique - Hôpitaux de Paris Paris France; 2 Service de Chirurgie Digestive Hôpital Européen Georges Pompidou Assistance Publique - Hôpitaux de Paris Paris France; 3 Université de Paris Paris France; 4 METHODS team Epidemiology and Biostatistics Sorbonne Paris Cité Center INSERM 1153 Paris France; 5 Service de Nutrition, Centre Spécialisé Obésité Hôpital Ambroise Paré Assistance Publique-Hôpitaux de Paris Boulogne-Billancour France; 6 Unité de recherche clinique Hôpital européen Georges Pompidou Assistance Publique-Hôpitaux de Paris Paris France; 7 Centre d’investigation clinique, INSERM 1418 Hôpital Européen Georges Pompidou Assistance Publique-Hôpitaux de Paris Paris France

**Keywords:** bariatric surgery, telemedicine, physical activity, RCT, gastric bypass, sleeve gastrectomy, connected pedometer, eHealth, mixed methods research

## Abstract

**Background:**

Despite bariatric surgery showing significant weight loss trajectories for many patients, a substantial proportion regain weight after the first year following surgery. The addition of telemedicine to standard care could support patients with engaging in a more active lifestyle and thus improve clinical outcomes.

**Objective:**

Our aim was to evaluate a telemedicine intervention program dedicated to the promotion of physical activity including digital devices, teleconsultation, and telemonitoring the first 6 months following bariatric surgery.

**Methods:**

This study employed a mixed methods design based on an open-label randomized controlled trial. Patients were included during the first week after bariatric surgery; then, they were randomized into 2 intervention groups: The TelePhys group received a monthly telemedicine consultation focusing on physical activity coaching, while the TeleDiet group received a monthly telemedicine consultation involving diet coaching. Data were collected using a watch pedometer and body weight scale, both of which were connected wirelessly. The primary outcome was the difference between the 2 groups in the mean numbers of steps at the first and sixth postoperative months. Weight change was also evaluated, and focus groups and interviews were conducted to enrich the results and capture perceptions of the telemedicine provided.

**Results:**

Among the 90 patients (mean age 40.6, SD 10.4 years; 73/90, 81% women; 62/90, 69% gastric bypass), 70 completed the study until the sixth month (n=38 TelePhys; n=32 TeleDiet), and 18 participants agreed to be interviewed (n=8 Telephys; n=10 TeleDiet). An increase in the mean number of steps between the first and sixth months was found in both groups, but this change was significant only in the TeleDiet group (*P*=.01). No difference was found when comparing both intervention groups. Interviewed participants reported having appreciated the teleconsultations, as the individualized tailored counseling helped them to make better choices about behaviors that could increase their likelihood of a daily life in better health. Weight loss followed by social factors (such as social support) were identified as the main facilitators to physical activity. Family responsibilities, professional constraints as well as poor urban policies promoting physical activity, and lack of accessibility to sport infrastructure were their major barriers to postoperative lifestyle adherence.

**Conclusions:**

Our study did not show any difference in mobility recovery after bariatric surgery related to a telemedicine intervention dedicated to physical activity. The early postoperative timing for our intervention may explain the null findings. eHealth interventions aiming to change behaviors and carried out by clinicians require support from structured public health policies that tackle patients’ obesogenic environment in order to be efficient in their struggle against sedentary lifestyle–related pathologies. Further research will need to focus on long-term interventions.

**Trial Registration:**

ClinicalTrials.gov NCT02716480, https://clinicaltrials.gov/ct2/show/NCT02716480

## Introduction

In patients living with severe obesity (BMI ≥40 kg/m^2^ or BMI ≥35 kg/m^2^ with ≥1 major obesity-related complication), bariatric surgery in combination with behavioral interventions is a more effective option than behavioral therapies alone for long-term weight loss (≥50% weight loss maintained for a minimum of 5 years) and remission of chronic conditions (such as type 2 diabetes, hypertension, sleep apnea, and dyslipidemia, as well as other conditions associated with increased adiposity) [1-3]. For 6 months to 12 months following surgery, patients usually experience a “honeymoon period,” when weight loss is drastic and rapid, mechanically driven by the modification of the digestive tract induced by surgery [4]. However, in order to improve weight loss and body composition and optimize health while minimizing postoperative complications, patients are recommended to actively adhere to a dietary protocol including vitamin and micronutrient intake and to engage in regular physical activity [5].

During the first postoperative weeks, patients transition from a liquid to soft to solid diet. They are encouraged to adopt new eating routines that consist of a low-fat, moderate-carbohydrate, and high-protein diet; small portions; 3 to 5 balanced and structured meals; and healthy snacks (chew food slowly and avoid sweets), as well as avoidance of carbonated beverages and caffeinated drinks, as the phosphoric acid and caffeine, respectively, can increase the risk of ulcerations [5]. In addition, early recovery to physical activity is also highly recommended: 30 minutes per day (150 minutes per week) of moderate-intensity exercise is associated with a slight additional weight loss, compared with patients who do not exercise after bariatric surgery [6]. Moreover, physical activity has been shown to enhance patients’ mood [7] and boost their motivation to sustain the recommended behavioral changes [5]. In the long term, higher physical activity can help promote further weight loss [8].

Despite bariatric surgery resulting in significant weight loss trajectories for many patients, a nonneglected proportion of patients regain weight after the first year following surgery [9]. There is a lack of consistent literature addressing predictors of weight regain after bariatric surgery. In addition to the surgical and anatomic factors that have been reported as significant predictors, other modifiable factors such as behavioral and psychosocial determinants need to be further investigated. Indeed, weight regain might be associated with poor adherence to postoperative dietary and physical activity recommendations [10]. As Gould et al [11] found, over two-thirds of patients miss appointments during the 2 first postoperative years, and only 40% meet their 4 annual recommended follow-up visits during the first year following gastric bypass surgery.

In order to improve long-term results and follow-up after surgery, the use of telemedicine or eHealth has been proposed for delivering health care to surgical patients [12-16]. The concept consists of the delivery of health-related services and information using telecommunication technologies or digital devices. The addition of telemedicine to standard care could support patients in changing their health behaviors and thus improve clinical outcomes.

As shown by 2 recent literature reviews [13,16], available literature on the use of eHealth interventions for bariatric surgery aftercare is poor, most being feasibility and pilot studies. However, the few available studies report positive impacts of telemedicine on eating behaviors [13,17], physical activity [18,19], and level of knowledge on nutrition and surgery [20]. Between the 2 reviews, a total of 6 randomized controlled trials (RCTs) were captured, 2 of them still in progress [21,22]. Of the completed RCTs, 2 found significant improvements in eating psychopathology using online modules and telephone support [17] and preoperative telephone-based cognitive behavioral therapy [23]. A third RCT assessing the influence of a videoconferencing-delivered psychoeducational postsurgery group intervention found positive outcomes on weight loss, health-related quality of life, and eating-related disorders in the subgroup of patients with depressive symptoms but not for the global sample [17,22]. A fourth RCT reported that an eHealth intervention that included iPad minis with the MyFitnessPal application added effectiveness for postsurgery weight loss maintenance when compared with standard care [15]. None of the studies reported any data on physical activity.

As observed by Creel et al [23], patients undergoing bariatric surgery face multiple challenges in engaging in regular physical activity. For most, this means moving beyond a long history of inactivity, many failed attempts of becoming more active, orthopedic limitations, and poor exercise tolerance. Thus, despite resulting in substantial weight loss and improved self-efficacy for exercise, surgery needs to be complemented with behavioral interventions [24]. In this regard, eHealth interventions with features including self-monitoring and personalized feedback given remotely by a counselor are proven to be more effective than standard eHealth programs for the prevention and treatment of overweight and obesity in adults [25]. Moreover, the meta-ethnographic systematic review by Robinson et al [26] examining the use of digital and mobile technologies with patients undergoing elective surgery highlighted the effectiveness of empowered patient-centered strategies with content-tailored interventions in supporting postsurgery health behavior change.

The paucity of studies examining how eHealth interventions can support patients to engage, as early as possible after their bariatric surgery, in an active and healthy lifestyle led to this investigation. Our study aimed to evaluate the effectiveness of a telemedicine intervention program including teleconsultation and telemonitoring with digital devices for the promotion of physical activity in the 6 first months following bariatric surgery.

## Methods

### Study Design

This study used a mixed methods design based on an open-label RCT. It integrated quantitative data collected by questionnaires and qualitative data through focus groups and in-depth individual interviews using a sequential “QUANT quali” temporality design (the quantitative component precedes the qualitative one, and it is predominant). This study combined triangulation (to converge results from different methods to validate interpretations) and complementarity (to reinforce, illustrate, or clarify the results of one method by those of another) [27]. Moreover, it applied an interpretative strategy for integrating the collected quantitative and qualitative data.

### Ethical Considerations

This study was conducted in accordance with the ethical standards of the institutional research committee and with the 1964 Helsinki Declaration and its later amendments or comparable ethical standards. This study was approved by the institutional medical ethical board Committee for the Protection of Persons of the Ile-de-France Region XI (approval number: IDRCB 2015-A01787-42/CPP 15053).

Informed consent was obtained from all individual participants included in the study.

The sponsor was My Goodlife SAS (Paris, France).

### Study Sample

Patients who underwent bariatric surgery (gastric bypass, sleeve, or adjustable gastric band) were recruited from 2 university teaching public hospitals in the Paris area (France) from June 2016 to July 2018, and data collection was completed in February 2019. Indications to perform surgeries relied on the guidelines published by the National Institute of Health [2]. Patients learned about the study at their last consultation with their surgeon before the operation. Those interested signed the informed consent and completed baseline questionnaires during the first postoperative week. Then, for each participant, telemedicine interventions began at 1 month postoperatively and lasted for 6 months. Inclusion criteria were as follows: age >18 years; preoperative weight less than 150 kg (due to the technical weight limit of the scale); primary surgery only; no postoperative complication between surgery and 1 month postoperative; valid home web access with computer, tablet, or smartphone; signed informed consent; ability to read and write in the French language; and valid affiliation with the French social security. All patients who did not meet the inclusion criteria were excluded.

### Randomization

**Figure 1:**
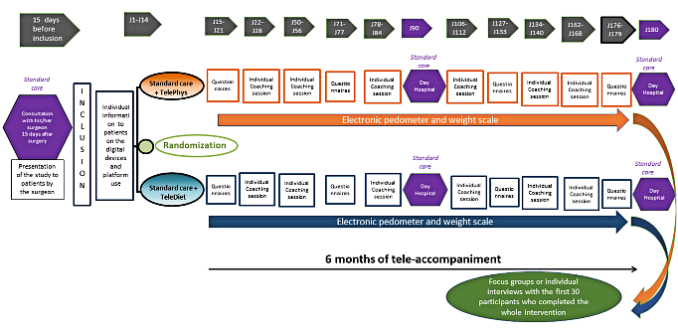
Flow chart of MyGoodTrip study design.

The randomization was computer-generated, and patients were allocated after they had signed the informed consent. To avoid patient dropouts in the control group, patients were randomized in 2 different and parallel intervention groups involving telemedicine: (1) remote physical activity coaching by phone led by a case manager with training in physical activity, diet, and behavior modification (TelePhys group) [28] or (2) remote dietary coaching by phone led by a dietitian (TeleDiet group) according to standard French guidelines for postbariatric follow-up [29]. The TeleDiet group was the control group for the TelePhys group and vice versa. At inclusion, each patient received 2 wireless telemonitoring devices: an electronic body weight scale (WebCoach POP CSI31360WH) and a pedometer band (Activi-T Band CTW41329BK) from Terraillon [30]. For both groups, the web platform tracked the parameters collected by the connected pedometer and scale, organized appointments with patients, and ensured traceability of exchanges with patients. The study design is presented in Figure 1.

### Consultations and Assessments

In France, the standard management of postoperative follow-up of patients having undergone bariatric surgery consists of giving the patient a prescription for outpatient diet and lifestyle advice by an ambulatory nutritionist doctor or dietitian trained to follow bariatric patients [29]. Patients operated at the hospitals in the study have consultations with their surgeon, a dietitian, and a teacher in adapted physical activity at the hospital at 1 month postsurgery. At 3 months and 6 months postoperatively, patients have a one-day hospital appointment with the multidisciplinary bariatric staff to undergo an assessment of comorbidities, laboratory tests for nutritional deficiencies, and investigation of eventual abnormal results. At these one-day hospital appointments, they also have individual consultations with nutritionist doctors and participate in 2 collective workshops, 1 with a dietitian and 1 with a trainer in adapted physical activity.

For 6 months, patients in the TelePhys group received a monthly one-to-one tele-accompaniment of 30 minutes. To enable personalized coaching, patients’ initial mobility diagnosis was based on the self-administered Recently Modified Physical Activity Questionnaire (RMPAQ) [31,32] and additional questions to determine the patient’s barriers and motivation to using different means of locomotion (car, walking, public transport, active transport) for home-to-work travel, daily utility travel, and leisure, as well as their barriers and motivation to change to more physically active means of locomotion. In particular, the RMPAQ consists of 9 questions covering 4 areas of physical activity: domestic life, work, leisure, and transportation [31]. It includes questions about the use of a computer, television viewing, and climbing stairs at home. The travel section concerns 4 usual modes of locomotion: walking, cycling, car, and public transport. The TelePhys coach acted as a case manager who followed the patient’s return to a more active lifestyle (engaging in a sport, doing regular exercise at home, dynamic walking or gardening, use of daily active modes of locomotion) in order to keep them motivated in their efforts, individually guide them in their daily travel choices, and help them solve problems. The TelePhys intervention was not based on a general outcome goal (such as reaching 10,000 steps per day or 150 minutes of structured exercise per week) that was communicated to patients. Instead, it was process-oriented, focusing on empowering and educating patients on self-monitoring while collaboratively setting personalized goals that reflected their aims, values, and current condition within the context of their environment (available equipment, resources, and social support).

The TeleDiet intervention also consisted of one-to-one coaching teleconsultation that lasted 30 minutes and was repeated monthly for 6 months. The dietitian who conducted them followed each patient’s diet and eating practices in order to provide them personalized advice on how to adjust to the postoperative dietary recommendations (namely, to multiply the daily meals, each being of a reduced volume; chew food well; limit drinks during meals; and avoid soft drinks).

The primary outcome of this study was the difference in the mean number of steps measured during a period of 14 days at the first and sixth postoperative months between the TelePhys and TeleDiet groups. Data were collected using the connected wireless watch pedometer. Weight change was analyzed during the 6 postoperative months using the connected weight scale and reported as weight in kilograms and percent weight loss.

A series of focus groups with 4 to 6 participants each was conducted at the end of patients’ participation in the TelePhys or TeleDiet intervention. For the patients who were not available or did not feel comfortable with speaking in a group, the option of an individual interview was given. Focus groups and interviews aimed to qualitatively capture the patients’ experiences with the telemedicine provided, their use of the connected watch pedometer and body weight scale, and the effects of the intervention on their lifestyle after bariatric surgery.

Drafts of the interview research guide were collaboratively designed by researchers and clinicians who worked with bariatric patients. Interview items were structured into 3 major thematic areas: (1) social representations on healthy eating practices and physical activity; (2) social conditions for lifestyle changes, adaptation, or inertia; and (3) assessment on how health digital devices and telemedicine helped them to implement nutritional prescriptions (“nutrition” covering both diet and physical activities). Participants were also asked to compare their presurgery and postsurgery experiences and to identify the factors and conditions that induced changes in their own thoughts, attitudes, and behaviors (see Multimedia Appendix 1 for the interview guide). Both qualitative techniques were conducted by a sociologist (KLiP) and lasted for about 60 minutes for the individual interviews and between 150 minutes and 200 minutes for the focus groups.

### Data Analysis

Statistical results were reported according to the criteria in the CONSORT statement. Intention-to-treat analysis was performed. Quantitative and qualitative variables were described by means (SDs) and frequencies (%). We tested for potential differences using analysis of variance or Kruskall-Wallis tests for continuous variables and Fisher exact tests for discrete variables. Paired *t* tests were used for intragroup comparisons. Data analyses were conducted using SAS version 9.4 (SAS Institute), and *P*<.05 was considered to indicate statistical significance.

Concerning the qualitative data from this mixed method research study, individual and group interviews were audio-recorded and transcribed verbatim. We performed a thematic content analysis with the support of ATLAS.ti 8.2.4 (ATLAS.ti.GmbH) software, coding the data with the constant comparative technique around the 3 major thematic areas (social representations, social conditions for lifestyle changes, and assessment using eHealth devices for adherence to nutritional prescription) and cross-checking for accuracy. According to our thematic focus on the promotion of physical activity, the analysis of the qualitative data presented here addressed (1) participants’ social representations of physical activity, (2) the barriers and facilitators they encountered while trying to decrease their sedentary lifestyle and increase their physical activity, and (3) their experiences with how digital devices (and exclusively for the patients from the TelePhys group, the teleconsultations) helped them to implement postoperative prescriptions for physical activity. Quotes in this paper were selected based on conceptual fit and according to how they captured the common perceptions and experiences expressed by more than one interviewee.

## Results

We randomized 90 participants to the TelePhys or TeleDiet group. Mean age was 40.6 (SD 10.4) years, and mean BMI was 43.4 (SD 5.6) kg/m^2^, with a vast majority of the sample as women (73/90, 81%). The most performed surgery was gastric bypass (62/90, 69%), followed by gastric lap band (17/90, 19%) and sleeve (11/90, 12%). Preoperative characteristics and type of surgery performed in the TelePhys and the TeleDiet groups are detailed in Table 1. Both intervention groups were comparable at inclusion.

**Figure 2:**
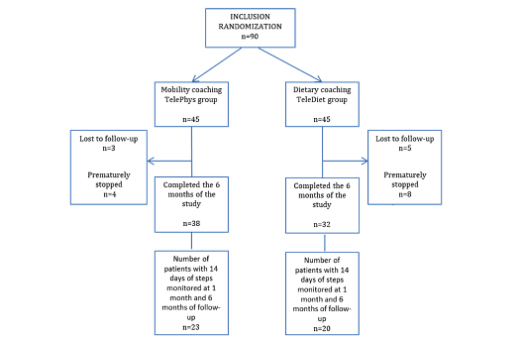
Flow chart of the MyGood Trip randomized trial.

Among the 90 patients enrolled, 70 completed the 6 months of the study (ie, a 22% participant dropout rate). The flow chart of the RCT is presented in Figure 2.

Among the patients in the TelePhys group (n=38) and the TeleDiet group (n=32) who completed the study, we obtained 14 days of numbers of steps from the connected pedometer watch during the first and sixth months from 23 (23/38, 61%) and 20 (20/32, 63%) patients, respectively.

We compared data from the participants who completed the 6-month assessment (Table 2) with those who did not (Table 3) in order to determine if there is differential attrition based on certain baseline characteristics: Patients who were lost to follow-up or stopped participating in the study were older than the patients who completed the 6-month assessment. There were also more patients who underwent gastric bypass among the TelePhys group and more men within the TeleDiet group who withdraw from participating in the study.

Both intervention groups had a difference in the number of steps per day during the 14-day period at the beginning of the study during the first month of monitoring, with the TelePhys group completing more steps (mean 7848.2, SD 3161.9 steps versus 6908.3, SD 2733.1 steps; *P*=.21; Table 4). An increase in the mean numbers of steps per day between the first and sixth months was found in both the TelePhys (mean 860.1, SD 3410.2 steps) and TeleDiet (mean 2419.7, SD 3647.0 steps) groups, but this difference was significant only in the TeleDiet group (*P*=.01). Moreover, even if the variation in steps count tended to be greater in the TeleDiet group, no difference was found in this change between the intervention groups (*P*=.17).

**Figure 3:**
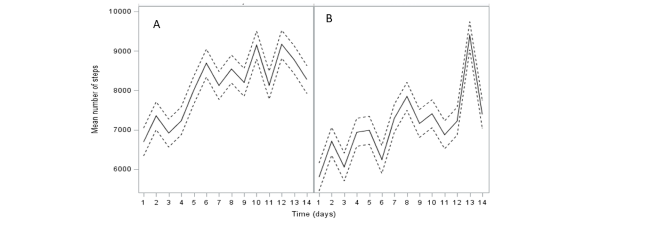
Mean number of daily steps evaluated with connected devices during the 6 months of the study in the (A) TelePhys and (B) TeleDiet groups.

Using the data on daily steps monitored by the connected band pedometer, Figure 3 shows the increase in the mean daily steps in both groups. A peak of physical activity was seen monthly in each intervention group corresponding to the days around a coaching teleconsultation.

As expected, significant weight loss was observed with bariatric surgery for both intervention groups at the sixth month (Table 5). No significant difference was found between the groups at the end of the intervention.

**Table 1:** Patients' characteristics at inclusion (n=90).

Variables			TelePhys group (n=45)		TeleDiet group (n=45)		Overall
Age (years), mean (SD)			41.9 (9.1)		39.4 (11.4)		40.6 (10.4)
Women, n (%)			38 (84)		35 (78)		73 (81)
BMI (kg/m^2^), mean (SD)^a^			42.7 (5.2)		44.0 (6.0)		43.4 (5.6)
Weight (kg), mean (SD)^a^			116.5 (15.8)		124.0 (16.6)		120.3 (16.5)
**Types of surgery, n (%)**							
	Gastric lap band	8 (18)		9 (20)		17 (19)	
	Gastric bypass	31 (69)		31 (69)		62 (69)	
	Sleeve	6 (13)		5 (11)		11 (12)	

^a^Preoperative data.

**Table 2:** Characteristics at inclusion of the patients who completed the 6-month assessment.

Variable		TelePhys group (n=38)	TeleDiet group (n=32)	Overall (n=90)
Age (years), mean (SD)		40.9 (9.3)	37.6 (10.5)	40.6 (10.4)
Women, n (%)		32 (84)	28 (88)	73 (81)
BMI (kg/m^2^), mean (SD)		43.0 (4.9)	43.9 (5.5)	43.4 (5.6)
Weight (kg), mean (SD)		116.6 (15.6)	123.1 (16.2)	120.3 (16.5)
**Types of surgery, n (%)**				
	Gastric lap band	7 (18)	5 (16)	17 (19)
	Gastric bypass	25 (66)	22 (69)	62 (69)
	Sleeve	6 (16)	5 (16)	11 (12)

**Table 3:** Characteristics at inclusion of the patients who were lost to follow-up or stopped participating in the study.

Variable		TelePhys group (n=7)		TeleDiet group (n=13)		Overall (n=90)
Age (years), mean (SD)		47.0 (6.4)		44.0 (12.7)		40.6 (10.4)
Women, n (%)		6 (86)		7 (54)		73 (81)
BMI (kg/m^2^), mean (SD)		41.2 (7.1)		44.2 (7.3)		43.4 (5.6)
Weight (kg), mean (SD)		116.4 (17.9)		126.5 (17.8)		120.3 (16.5)
**Types of surgery, n (%)**						
	Gastric lap band	1 (14)	4 (31)		17 (19)	
	Gastric bypass	6 (86)	9 (69)		62 (67)	
	Sleeve	0 (0)	0 (0)		11 (12)	

**Table 4:** Mean numbers of steps per day during the 14-day period of monitoring at the first and sixth postoperative months in the TelePhys and TeleDiet groups.

Variable	TelePhys group	TeleDiet group	*P* value
Number of steps at month 1, mean (SD)	7848.2 (3161.9)^a^	6908.3 (2733.1)^b^	.21
Numbers of steps at month 6, mean (SD)	9294.7 (3234.3)^c^	9764.3 (4268.8)^d^	.68
Difference (month 6-month 1), mean (SD)	860.1 (3410.2)^e^	2419.7 (3647.0)^f^	.17
Intragroup comparison, *P* value	.25	.01	N/A^g^

^a^n=31.

^b^n=32.

^c^n=23.

^d^n=20.

^e^n=22.

^f^n=19.

^g^N/A: not applicable.

**Table 5:** Changes in body weight monitored with the connected weight scale at the first, third, and sixth postoperative months in the TelePhys and TeleDiet groups.

Variable	TelePhys group	TeleDiet group	*P* value	
Weight at month 1 (kg), mean (SD)	103.1 (16.5)^a^	111.7.0 (17.9)^a^	.08	
Weight at month 3 (kg), mean (SD)	98.8 (15.7)^b^	101.6 (16.8)^b^	.52	
Weight at month 6 (kg), mean (SD)	86.8 (16.0)^c^	89.4 (15.1)^d^	.50	
Difference (month 6-month 1; kg), mean (SD)	–16.6 (8.1)^e^	–19.3 (7.0)^f^	.26	
Intragroup comparison, *P* value	<.001	<.001	N/A^g^	

^a^n=29.

^b^n=28.

^c^n=31.

^d^n=24.

^e^n=22.

^f^n=19.

^g^N/A: not applicable.

Due to research budget restrictions, the qualitative part of the study could only be proposed to the first one-half of patients who completed the 6-month intervention (n=35). A total of 18 participants agreed to be interviewed (8 patients in the TelePhys group and 10 patients in the TeleDiet group); 3 focus groups of 4 participants each and 6 individual interviews were finally conducted. Among the TelePhys patients who participated in the qualitative part of the study, one-half opted for an individual interview (4/8, 50%), and the other one-half opted for the focus group (4/8, 50%). Among the 10 TeleDiet patients, 2 chose the individual interview, and 8 participated in a focus group.

For all these participants, “physical activity” was first associated with “sports,” defined as intense, tiring exercises that are practiced in leisure time, with the guidance of a sports instructor.

When we are told “you need to do more physical activity,” I directly think about going to the gym.

P6, TelePhys, interview

For me, it’s getting back into physical condition, at the level of endurance and breathing again, a kind of little punch. All the things I didn’t have before. (...) So, I started to go to the fitness room 3 weeks ago.

P12, TeleDiet, interview

Patients in the TelePhys group also mentioned day-to-day activities requiring bodily efforts such as “household,” “gardening,” “taking the stairs instead of the lift,” and “preferring walking to the bus,” actions that they understand better as “mobility activities.”

Now I know that everything that is daily activity (cleaning etc) is considered physical activity, but this is not the first thing I think about as “physical activity” but as “mobility,” namely all that can increase my physical activity in everyday life: to prefer walking to the bus, stairs to elevators, in order to try to move more.

P1, TelePhys, interview

If sport was viewed as producing suffering and discomfort, mobility practices such as walking or biking for their home or work travels were seen as a source of mental wellness.

Physical activity? It’s gym, pain, sweat [laughter shared by all], stuff that hurts, that doesn’t make you want to.

P5, TelePhys, focus group

When I go home, I’m in my head. I have my music. I want to think about something else, to evacuate, so I get off the bus some stops earlier. It’s true; it feels good.

P4, TelePhys, interview

I have just moved to a city that is more suitable for bicycle transport (...), and it feels so good going back home by bike.

P6, Telephys, focus group

Despite their weight loss and willingness, participants expressed difficulties in integrating higher and sustained physical activity in the very context of their day-to-day lives. The 3 factors most commonly mentioned as jeopardizing their efforts to decrease their sedentary lifestyle were (1) professional constraints, in particular extended working hours and sedentary office work; (2) the long Paris-suburban areas of home-to-work travel time; and (3) lack of urban policies that would promote outdoor activities with either practicable green areas, safe pedestrian walkways, and bike path network and investment in sport infrastructure that is accessible by everyone.

I’m working at a bank (...) in a high responsibility working position. There are weeks of hyperintensive work. These last few weeks, I have been going to work at 7 AM and coming home at 11 PM (...) This weekend, I also stayed for long hours working on the computer.

P17, TeleDiet, interview

From home to my office, I put in 1 hour 20 minutes. Public transport is so direct that I barely walk. Of course, I could try to get off earlier and continue on foot, but it is really my schedule that...because I leave very early from home and come back late. Also, to be honest, my neighborhood doesn’t make you want to walk more, especially when it gets dark.

P4, TelePhys, focus group

I tried cycling in Paris. It was one of my goals to lose weight. But, take the bike paths: There aren’t many, they’re on the sidewalk, so they’re overrun by pedestrians. It’s a 2-way street, so people sometimes go from right to left. Then, the bus corridors: It’s not bad, but on the security level, it’s still to be seen. Buses are aware, pay attention, but taxis that use the bus tracks are not. Well, too stressful, a real danger.

P7, TelePhys, focus group

In our neighborhood, parks are not clean. There are drunk people or taking drugs or whatever, so not a very pleasant environment for a promenade.

P8, TelePhys, focus group

Self-presentational concerns were also mentioned by a few patients, who also expressed how establishing new routines takes time.

I love dancing, but I really feel the need to lose more weight to take dance classes. Well, it’s in my head, I know. I can’t avoid thinking that it’s maybe more beautiful to see someone slimmer who dances than someone round shaped. [Interviewer: Where are you now on this project?] Maybe, uh, a little more concrete. I visualize it a bit better, but maybe I’ll wait a little longer for me to feel better.

P2, TelePhys, interview

The primary facilitator for engaging in a sport activity was weight loss, not only because it decreased pain and physical strain but also because it was felt as a response to the fatigue brought by rapid and intensive body mass loss.

I felt quite weak because of my weight loss, and I still feel it now. So, I started to go to the gym to regain a little of strength and energy.

P12, TeleDiet, focus group

Close friends, family members, and work colleagues were also highlighted as important support in their lifestyle change, especially in making them discover new sports.

I started cycling with my daughters and wife on weekends in the countryside, next to Paris.

P5, TelePhys, interview

I do aquagym on Thursday evenings. A cousin was attending the course, and I joined her.

P1, TelePhys, interview

Near my office, there is a sports club doing things in the evenings. (...) I have a coworker who was doing aquagym there, and now we go together.

P9, TeleDiet, focus group

In general, patients considered digital health devices as useful tools to help them start implementing postoperative prescriptions for physical activity. Self-tracking of their steps, weight loss, or fat-muscle rebalancing in the present time was highlighted as a method that allowed them to objectify their physical changes. Digital devices brought them well-defined benchmarks to inform them on their efforts on a daily basis.

It is very encouraging when you see the curves of weight loss and of the changes in the body mass composition, what has changed inside of your body in terms of fat, water, muscle, etc.

P16, TeleDiet, focus group

We know that we’re losing weight and body mass, but to be able to see the fat loss or muscle gain, I find it very nice. It motivated me to go on.

P1, TelePhys, interview

I used to check the watch pedometer more at the end of the day when coming back home: “I did that many steps so far”; it gave me an indicator, “Okay, I’ll walk a little more.” So, I’ll get off at a few stations before with the idea of increasing the number of steps to reach at least my daily goal of 10,000 steps.

P5, TelePhys, focus group

TelePhys patients considered the teleconsultations as a helpful complementary tool for their postsurgery follow-up to strengthen their adherence to postoperative recommendations. We drew 3 major reasons from their feedback: (1) It boosted their motivation; (2) it gave specific information that reassured them regarding their intense, surgery-driven body changes; and (3) it provided tailored advice according to their socioeconomic situation and changing physical and psychological needs.

The regular contact with the coach helped me to keep motivated to change my eating habits and start moving more.

P1, TelePhys, interview

Having an expert with whom to speak regularly by phone about my situation was reassuring. (...) Even if the medical team had previously informed us about the weight loss process and food changes after the surgery, these are actually hard to go through once back home.

P4, TelePhys, interview

The coach asked me what I was doing in my real life so we could work together on how to customize.

P7, TelePhys, focus group

To have someone who is there and who really adapts things to our case and who does not give courses that are a little more general.

P2, TelePhys, interview

It gave immediate response to the problems and difficulties I was going through to be more active.

P5, TelePhys, focus group

## Discussion

### Principal Findings

This RCT was designed to examine the effects on sedentary behavior and weight loss of an intervention that combined the use of eHealth technologies (pedometer and scale) and physical activity coaching by telemedicine with patients who had undergone bariatric surgery, compared with an intervention that combined the use of the same digital devices and dietary counseling by telemedicine, during the first 6 months after bariatric surgery. As expected, an increase in the mean number of steps and weight loss between the first and sixth months were observed in both intervention groups, but no significant difference was found when comparing these changes between the groups.

During the postoperative period, rapid, substantial weight loss is mechanically induced by surgery. Previous studies have shown that the strong physiological effects of surgery were a major reason for a lack of a significant effect of complementary behavioral interventions [6,33]. During this “honeymoon period,” most patients may have also considered being more active as unnecessary, in particular when exercise was only perceived as a means to lose weight [24]. Moreover, given the higher physical activity doses recommended for weight loss, the average steps or exercise regularly performed by most participants was possibly insufficient to enhance the weight loss produced by surgery [19,34,35]. The fact that all participants had connected scales and pedometers to get objective measures of their physical and behavioral changes could have canceled the differential effects of the physical activity counseling provided in the TelePhys intervention. Despite receiving tailored counseling, the timing of the research may have also been insufficient for the participants in the TelePhys group to overcome the barriers of physical activity, most of which were not obesity-related.

As the qualitative study highlighted, professional and family constraints and lack of urban and transport policy promoting physical activity may help to partly explain our null results. According to previous studies, weight loss is commonly reported as the most important facilitator to physical activity, as it directly reduces lower limb and back pain, joint load, and physical strain, as well as obesity-related fatigue and flabbiness [24,35,36]. However, social factors play a major role in developing a sustainable, long-term habit of being physically active. Having social support in the closest environment, such as having company when engaging in physical activity or someone to take over part of the patient’s household activities or family care work while exercising, has also been highlighted as an important aspect of starting and maintaining physical activity [24,36]. Our Parisian population mainly reported family responsibilities and professional constraints (eg, work schedule, sedentary office work, long work-to-home travel), as also found in the literature [34,35]; poor urban policies promoting outdoor or indoor physical activities that are accessible for all (unpractical green areas, unsafe pedestrian walkways and bike path network); and lack of accessibility of sport infrastructure (nearby gym or public swimming) as major barriers to physical activity.

The 77% of participants who completed the 6-month intervention period despite technological problems with the connected devices (22% of participants stopped the study mostly because of equipment malfunction) led us to support the feasibility of proposing a combination of eHealth devices and telemedicine along with standard postsurgery follow-up. As observed in the qualitative study, digital devices were appreciated, as they allowed an assessment of participants’ behavior and physical changes in real time and within their own daily environment. They provided tangible benchmarks to inform the patients on the effort to be made each day. In doing so, eHealth technology helped patients gain autonomy in their long-term postoperative care [23]. As regards the teleconsultations, participants in the qualitative study highlighted that monthly, one-to-one teleconsultations helped them overcome their personal barriers and raise unmet individual needs beyond routine clinical questioning [37], which helped them gain confidence and increase their feelings of accountability and responsibility regarding their postoperative care. Specific to the TelePhys group, exchanges with the case manager allowed them to consolidate a renewed perception of what constitutes physical activity (more as routine daily life practices that decrease their sedentary behavior) and develop strategies and take action to engage in a more active lifestyle [35]. In line with previous studies [16,26], participants also felt that the individualized tailored counseling helped them to make better choices, in particular as teleconsultations gave personalized knowledge on the behaviors that could increase the likelihood of a daily life in better health and addressed the ways to overcome the concerned participant’s difficulties with postoperative lifestyle adherence.

This study is the first multicentric RCT that combined digital devices (connected wireless pedometer and scale) and telemedicine (on physical activity or diet counseling) to strengthen patients’ empowerment to play an active role in their long-term care after bariatric surgery. Interventions combined education with behavioral patient-centered strategies and were delivered in a collaborative way that respected patient autonomy. This RCT used a complementary qualitative approach to reinforce, illustrate, or clarify results. Mixed methods research helps capture the complex influences of individual and contextual processes [38]. Our results add knowledge to the current debates on the design of optimal physical activity interventions needed to optimize the results of bariatric surgery.

Due to time constraints of the study funding, we were unable to follow the patients for a longer period of time and increase the sample of participants in the qualitative study. With a longer follow-up, the results would have provided a deeper understanding of the ways in which digital technologies and telemedicine can support patients in the development of healthier behavior changes and their routinization. However, the timing of the RCT covered the earliest process of behavioral changes after the surgery, which is more a phase of understanding and coping with surgery-induced body changes and of gaining awareness of the importance of being engaged in an active lifestyle for the bariatric surgery to have a greater impact on their health and life conditions. Physical coaching via telemedicine for patients having undergone bariatric surgery needs to be analyzed with a longer follow-up to not only remove biases created by the immediate and impressive effect of weight loss but also better measure its impact on the routinization of health behavior changes. Most TelePhys participants were, at the moment of the intervention, rediscovering their body and new physical abilities and exploring new sports activities or renewing activities practiced in the past. They also were in the middle of a phase of reviewing their use of time and reorganization of their family and work lives to make more room for physical activity. Then, the ideal study timing would have cover at least 1 year after weight stabilization. Moreover, it may also have been more relevant to measure a decrease in sedentary behavior with wearable activity tracking monitors and sensors than only focusing on increasing steps [19,39]. All studies using connected devices face the limits of participants’ digital competence, which is an issue that should be addressed in future clinical research to make eHealth interventions adjunct to standard postsurgical care accessible for all. The randomization of the trial could also have contributed to patient dropout by assigning certain patients to the intervention group that addressed a need already satisfied. As stated earlier and elsewhere [16,26,40-42], eHealth interventions need to be tailored to the patient’s socioeconomic situation and changing physical needs.

### Conclusion

The MyGoodTrip RCT was not able to show a statistically significant effect of a telemedicine intervention dedicated to physical activity in helping patients gain a more active lifestyle after bariatric surgery. The early postoperative time frame for our intervention may explain the null findings. However, in the light of its qualitative findings, this study highlighted both the need (expressed by the participants in the TelePhys intervention) and challenge for health care professionals to provide patients, after bariatric surgery, with individually tailored information about the benefits of physical activity and advice to empower them to overcome their obesity-related and motivational barriers, so that these patients can find the right attitude and make adequate decisions to engage in sustainable physical activity. This study also highlighted how behavioral change-seeking eHealth interventions conducted by clinicians require support from structured public health policies to tackle patients’ obesogenic environments in order to be efficient in their struggle against sedentary lifestyle–related pathologies.

Further research using an interdisciplinary, mixed method approach will need to focus on long-term eHealth interventions adjunct to standard bariatric postoperative clinical care, tailored according to the patient’s socioeconomic situation and changing physical needs.

## Appendix

Multimedia Appendix 1 Focus group interview guide.

Multimedia Appendix 2 CONSORT-eHEALTH checklist (V 1.6.1).

## Abbreviations

RCT: randomized controlled trial
RMPAQ: Recently Modified Physical Activity Questionnaire
