# Supplementary material for: MyGood Trip, a Telemedicine Intervention for Physical Activity Recovery After Bariatric Surgery: Randomized Controlled Trial
Source: JMIR Form Res. 2023 Mar 28;7:e26077. doi: 10.2196/26077 (PMC10132008; doi:10.2196/26077)
Supplement: Multimedia Appendix 1 [file formative_v7i1e26077_app1.docx]

**MyGoodTrip Randomized Control Trial**

**Focus group and semistructured one-on-one interview guide**

**Part 1. Social representations on “physical activity”**

What does “physical activity” mean to you?

How would you define yourself according the physical activity you do today?

What do you consider as “sedentary”?

How would you define yourself in terms of sedentary behavior?

What sensations of pleasure or displeasure do you associate with the following means of travel: walking, public transport, cycling, scooter, car and carpooling? Could you develop?

**Part 2. Social conditions of physical activity behavior changes following the bariatric surgery**

Before the surgery, did you set specific physical activity goals for yourself?

Since the surgery, have you made anything to be physically more active?

How did you come up with such changes? What do you think were the main reasons for them? What made them possible?

On the contrary, what have impeded you be more physically active / reach your physical activity goals?

Have changes in your physical activity and travel patterns had an impact on the organization of your day-to-day life? What accommodations have you made in your personal, family and working life?

And what about your close family members: have you observed any changes in their physical activities? How can explain such changes?

**Part 3. Participant’s experience with the e-health devices and the teleconsultations**

Regarding the digital devices, what did you find particularly interesting? What did you like the most? What about the teleconsultations?

Regarding the digital devices, were there any difficult thing or anything that disappointed you? What about the teleconsultations?

To what extent has your participation at this interventional study contributed to changes in your physical activity? How did you experience such changes? What do you think of them?

What changes would you make to the intervention program into which you took part in order to improve the post-operative follow-up?
