# Supplementary material for: MyGood Trip, a Telemedicine Intervention for Physical Activity Recovery After Bariatric Surgery: Randomized Controlled Trial
Source: JMIR Form Res. 2023 Mar 28;7:e26077. doi: 10.2196/26077 (PMC10132008; doi:10.2196/26077)
Supplement: Multimedia Appendix 2 [file formative_v7i1e26077_app2.pdf]

CONSORT-EHEALTH (V 1.6.1) - Submission/Publication Form

The CONSORT-EHEALTH checklist is intended for authors of randomized trials evaluating web-based and Internet-based applications/interventions, including mobile interventions, electronic games (incl multiplayer games), social media, certain telehealth applications, and other interactive and/or networked electronic applications. Some of the items (e.g. all subitems under item 5 - description of the intervention) may also be applicable for other study designs.

The goal of the CONSORT EHEALTH checklist and guideline is to be
a) a guide for reporting for authors of RCTs,
b) to form a basis for appraisal of an ehealth trial (in terms of validity)

CONSORT-EHEALTH items/subitems are MANDATORY reporting items for studies published in the Journal of Medical Internet Research and other journals / scientific societies endorsing the checklist.

Items numbered 1., 2., 3., 4a., 4b etc are original CONSORT or CONSORT-NPT (non-pharmacologic treatment) items.
Items with Roman numerals (i., ii, iii, iv etc.) are CONSORT-EHEALTH extensions/clarifications.

As the CONSORT-EHEALTH checklist is still considered in a formative stage, we would ask that you also RATE ON A SCALE OF 1-5 how important/useful you feel each item is FOR THE PURPOSE OF THE CHECKLIST and reporting guideline (optional).

Mandatory reporting items are marked with a red *.
In the textboxes, either copy & paste the relevant sections from your manuscript into this form - please include any quotes from your manuscript in QUOTATION MARKS,
or answer directly by providing additional information not in the manuscript, or elaborating on why the item was not relevant for this study.

YOUR ANSWERS WILL BE PUBLISHED AS A SUPPLEMENTARY FILE TO YOUR PUBLICATION IN JMIR AND ARE CONSIDERED PART OF YOUR PUBLICATION (IF ACCEPTED).
Please fill in these questions diligently. Information will not be copyedited, so please use proper spelling and grammar, use correct capitalization, and avoid abbreviations.

DO NOT FORGET TO SAVE AS PDF _AND_ CLICK THE SUBMIT BUTTON SO YOUR ANSWERS ARE IN OUR DATABASE !!!

Citation Suggestion (if you append the pdf as Appendix we suggest to cite this paper in the caption):
Eysenbach G, CONSORT-EHEALTH Group
CONSORT-EHEALTH: Improving and Standardizing Evaluation Reports of Web-based and Mobile Health Interventions
J Med Internet Res 2011;13(4):e126

URL: http://www.jmir.org/2011/4/e126/
doi: 10.2196/jmir.1923
PMID: 22209829

katia.lurbe.puerto@gmail.com (non partagé) Changer de compte

Brouillon non enregistré

*Obligatoire

Your name *

First Last

Katia LURBE PUERTO

Primary Affiliation (short), City, Country *

University of Toronto, Toronto, Canada

Service de Nutrition, Centre Spécialisé Obésité

Your e-mail address *

abc@gmail.com

katia.lurbe.puerto@gmail.com

Title of your manuscript *

Provide the (draft) title of your manuscript.

MyGood Trip, a Telemedicine Intervention for Physical Activity Recovery After Bariatric Surgery: Randomized Controlled Trial

Name of your App/Software/Intervention *

If there is a short and a long/alternate name, write the short name first and add the long name in brackets.

electronic body weight scale (WebCoach POP

Evaluated Version (if any)

e.g. "V1", "Release 2017-03-01", "Version 2.0.27913"

WebCoach POP CSI31360WH and Activi-T Ban

Language(s) *

What language is the intervention/app in? If multiple languages are available, separate by comma (e.g. "English, French")

French, English

URL of your Intervention Website or App

e.g. a direct link to the mobile app on app in appstore (itunes, Google Play), or URL of the website. If the intervention is a DVD or hardware, you can also link to an Amazon page.

Votre réponse

URL of an image/screenshot (optional)

Votre réponse

Accessibility *

Can an enduser access the intervention presently?

- ○ access is free and open
- ○ access only for special usergroups, not open
- ◉ access is open to everyone, but requires payment/subscription/in-app purchases
- ○ app/intervention no longer accessible
- ○ Autre :

Primary Medical Indication/Disease/Condition *

e.g. "Stress", "Diabetes", or define the target group in brackets after the condition, e.g. "Autism (Parents of children with)", "Alzheimers (Informal Caregivers of)"

Obesity ; Diabetes ; Post-bariatric surgery inter

Primary Outcomes measured in trial *

comma-separated list of primary outcomes reported in the trial

number of steps, weight change, user's percep

Secondary/other outcomes

Are there any other outcomes the intervention is expected to affect?

Votre réponse

Recommended "Dose" *

What do the instructions for users say on how often the app should be used?

- (x) Approximately Daily
- ( ) Approximately Weekly
- ( ) Approximately Monthly
- ( ) Approximately Yearly
- ( ) "as needed"
- ( ) Autre :

Approx. Percentage of Users (starters) still using the app as recommended after 3 months *

- (x) unknown / not evaluated
- ( ) 0-10%
- ( ) 11-20%
- ( ) 21-30%
- ( ) 31-40%
- ( ) 41-50%
- ( ) 51-60%
- ( ) 61-70%
- ( ) 71%-80%
- ( ) 81-90%
- ( ) 91-100%
- ( ) Autre :

Overall, was the app/intervention effective? *

- ( ) yes: all primary outcomes were significantly better in intervention group vs control
- ( ) partly: SOME primary outcomes were significantly better in intervention group vs control
- (x) no statistically significant difference between control and intervention
- ( ) potentially harmful: control was significantly better than intervention in one or more outcomes
- ( ) inconclusive: more research is needed
- ( ) Autre :

Article Preparation Status/Stage *

At which stage in your article preparation are you currently (at the time you fill in this form)

- ( ) not submitted yet - in early draft status
- ( ) not submitted yet - in late draft status, just before submission
- ( ) submitted to a journal but not reviewed yet
- ( ) submitted to a journal and after receiving initial reviewer comments
- (x) submitted to a journal and accepted, but not published yet
- ( ) published
- ( ) Autre :

Journal *

If you already know where you will submit this paper (or if it is already submitted), please provide the journal name (if it is not JMIR, provide the journal name under "other")

- ○ not submitted yet / unclear where I will submit this
- ○ Journal of Medical Internet Research (JMIR)
- ○ JMIR mHealth and UHealth
- ○ JMIR Serious Games
- ○ JMIR Mental Health
- ○ JMIR Public Health
- ◉ JMIR Formative Research
- ○ Other JMIR sister journal
- ○ Autre :

Is this a full powered effectiveness trial or a pilot/feasibility trial? *

- ○ Pilot/feasibility
- ◉ Fully powered

Manuscript tracking number *

If this is a JMIR submission, please provide the manuscript tracking number under "other" (The ms tracking number can be found in the submission acknowledgement email, or when you login as author in JMIR. If the paper is already published in JMIR, then the ms tracking number is the four-digit number at the end of the DOI, to be found at the bottom of each published article in JMIR)

- ○ no ms number (yet) / not (yet) submitted to / published in JMIR
- ◉ Autre : JMR ms#26077

TITLE AND ABSTRACT

1a) TITLE: Identification as a randomized trial in the title

1a) Does your paper address CONSORT item 1a? *

I.e does the title contain the phrase "Randomized Controlled Trial"? (if not, explain the reason under "other")

- (x) yes
- ( ) Autre :

1a-i) Identify the mode of delivery in the title

Identify the mode of delivery. Preferably use “web-based” and/or “mobile” and/or “electronic game” in the title. Avoid ambiguous terms like “online”, “virtual”, “interactive”. Use “Internet-based” only if Intervention includes non-web-based Internet components (e.g. email), use “computer-based” or “electronic” only if offline products are used. Use “virtual” only in the context of “virtual reality” (3-D worlds). Use “online” only in the context of “online support groups”. Complement or substitute product names with broader terms for the class of products (such as “mobile” or “smart phone” instead of “iphone”), especially if the application runs on different platforms.

subitem not at all important

- 1 (x)
- 2 ( )
- 3 ( )
- 4 ( )
- 5 ( )

essential

Effacer la sélection

Does your paper address subitem 1a-i? *

Copy and paste relevant sections from manuscript title (include quotes in quotation marks "like this" to indicate direct quotes from your manuscript), or elaborate on this item by providing additional information not in the ms, or briefly explain why the item is not applicable/relevant for your study

It was delivered by the technical support staff of the methods team.

1a-ii) Non-web-based components or important co-interventions in title

Mention non-web-based components or important co-interventions in title, if any (e.g., “with telephone support”).

subitem not at all important

- 1 (selected)
- 2
- 3
- 4
- 5

essential

Effacer la sélection

Does your paper address subitem 1a-ii?

Copy and paste relevant sections from manuscript title (include quotes in quotation marks "like this" to indicate direct quotes from your manuscript), or elaborate on this item by providing additional information not in the ms, or briefly explain why the item is not applicable/relevant for your study

We used Telemedecine intervention which covers different e-health devices and telephone support

1a-iii) Primary condition or target group in the title

Mention primary condition or target group in the title, if any (e.g., “for children with Type I Diabetes”) Example: A Web-based and Mobile Intervention with Telephone Support for Children with Type I Diabetes: Randomized Controlled Trial

subitem not at all important

1 ○

2 ○

3 ○

4 ○

5 ◉

essential

Effacer la sélection

Does your paper address subitem 1a-iii? *

Copy and paste relevant sections from manuscript title (include quotes in quotation marks "like this" to indicate direct quotes from your manuscript), or elaborate on this item by providing additional information not in the ms, or briefly explain why the item is not applicable/relevant for your study

Yes : after bariatric surgery

1b) ABSTRACT: Structured summary of trial design, methods, results, and conclusions

NPT extension: Description of experimental treatment, comparator, care providers, centers, and blinding status.

1b-i) Key features/functionalities/components of the intervention and comparator in the METHODS section of the ABSTRACT

Mention key features/functionalities/components of the intervention and comparator in the abstract. If possible, also mention theories and principles used for designing the site. Keep in mind the needs of systematic reviewers and indexers by including important synonyms. (Note: Only report in the abstract what the main paper is reporting. If this information is missing from the main body of text, consider adding it)

subitem not at all important

- 1 (selected)
- 2
- 3
- 4
- 5

essential

Effacer la sélection

Does your paper address subitem 1b-i? *

Copy and paste relevant sections from the manuscript abstract (include quotes in quotation marks "like this" to indicate direct quotes from your manuscript), or elaborate on this item by providing additional information not in the ms, or briefly explain why the item is not applicable/relevant for your study

We are not concerned with this

1b-ii) Level of human involvement in the METHODS section of the ABSTRACT

Clarify the level of human involvement in the abstract, e.g., use phrases like “fully automated” vs. “therapist/nurse/care provider/physician-assisted” (mention number and expertise of providers involved, if any). (Note: Only report in the abstract what the main paper is reporting. If this information is missing from the main body of text, consider adding it)

subitem not at all important

1 ○

2 ○

3 ◉

4 ○

5 ○

essential

Effacer la sélection

Does your paper address subitem 1b-ii?

Copy and paste relevant sections from the manuscript abstract (include quotes in quotation marks "like this" to indicate direct quotes from your manuscript), or elaborate on this item by providing additional information not in the ms, or briefly explain why the item is not applicable/relevant for your study

"Patients were included during the first week after bariatric surgery; then, they were randomized into 2 intervention groups: The TelePhys group received a monthly telemedicine consultation focusing on physical activity coaching, while the TeleDiet group received a monthly telemedicine consultation involving diet coaching. Data were collected using a watch pedometer and body weight scale, both of which were connected wirelessly."

1b-iii) Open vs. closed, web-based (self-assessment) vs. face-to-face assessments in the METHODS section of the ABSTRACT

Mention how participants were recruited (online vs. offline), e.g., from an open access website or from a clinic or a closed online user group (closed usergroup trial), and clarify if this was a purely web-based trial, or there were face-to-face components (as part of the intervention or for assessment). Clearly say if outcomes were self-assessed through questionnaires (as common in web-based trials). Note: In traditional offline trials, an open trial (open-label trial) is a type of clinical trial in which both the researchers and participants know which treatment is being administered. To avoid confusion, use “blinded” or “unblinded” to indicated the level of blinding instead of “open”, as “open” in web-based trials usually refers to “open access” (i.e. participants can self-enrol). (Note: Only report in the abstract what the main paper is reporting. If this information is missing from the main body of text, consider adding it)

subitem not at all important

1 ○

2 ○

3 ○

4 ○

5 ◉

essential

Effacer la sélection

Does your paper address subitem 1b-iii?

Copy and paste relevant sections from the manuscript abstract (include quotes in quotation marks "like this" to indicate direct quotes from your manuscript), or elaborate on this item by providing additional information not in the ms, or briefly explain why the item is not applicable/relevant for your study

"This study employed a mixed methods design based on an open-label randomized controlled trial"

1b-iv) RESULTS section in abstract must contain use data

Report number of participants enrolled/assessed in each group, the use/uptake of the intervention (e.g., attrition/adherence metrics, use over time, number of logins etc.), in addition to primary/secondary outcomes. (Note: Only report in the abstract what the main paper is reporting. If this information is missing from the main body of text, consider adding it)

subitem not at all important

1 ○

2 ○

3 ○

4 ○

5 ◉

essential

Effacer la sélection

Does your paper address subitem 1b-iv?

Copy and paste relevant sections from the manuscript abstract (include quotes in quotation marks "like this" to indicate direct quotes from your manuscript), or elaborate on this item by providing additional information not in the ms, or briefly explain why the item is not applicable/relevant for your study

"Among the 90 patients (mean age 40.6, SD 10.4 years; 73/90, 81% women; 62/90, 69% gastric bypass), 70 completed the study until the sixth month (n=38 TelePhys; n=32 TeleDiet), and 18 participants agreed to be interviewed (n=8 Telephys; n=10 TeleDiet)."
"technological problems with the connected devices (22% of participants stopped the study mostly because of equipment malfunction)"

1b-v) CONCLUSIONS/DISCUSSION in abstract for negative trials

Conclusions/Discussions in abstract for negative trials: Discuss the primary outcome - if the trial is negative (primary outcome not changed), and the intervention was not used, discuss whether negative results are attributable to lack of uptake and discuss reasons. (Note: Only report in the abstract what the main paper is reporting. If this information is missing from the main body of text, consider adding it)

subitem not at all important

- 1 ○
- 2 ○
- 3 ○
- 4 ○
- 5 ◉

essential

Effacer la sélection

Does your paper address subitem 1b-v?

Copy and paste relevant sections from the manuscript abstract (include quotes in quotation marks "like this" to indicate direct quotes from your manuscript), or elaborate on this item by providing additional information not in the ms, or briefly explain why the item is not applicable/relevant for your study

"Our study did not show any difference in mobility recovery after bariatric surgery related to a telemedicine intervention dedicated to physical activity. The early postoperative timing for our intervention may explain the null findings. eHealth interventions aiming to change behaviors and carried out by clinicians require support from structured public health policies that tackle patients’ obesogenic environment in order to be efficient in their struggle against sedentary lifestyle–related pathologies. Further research will need to focus on long-term interventions."

INTRODUCTION

2a) In INTRODUCTION: Scientific background and explanation of rationale

2a-i) Problem and the type of system/solution

Describe the problem and the type of system/solution that is object of the study: intended as stand-alone intervention vs. incorporated in broader health care program? Intended for a particular patient population? Goals of the intervention, e.g., being more cost-effective to other interventions, replace or complement other solutions? (Note: Details about the intervention are provided in "Methods" under 5)

subitem not at all important

- 1 ○
- 2 ○
- 3 ○
- 4 ○
- 5 ◉

essential

Effacer la sélection

Does your paper address subitem 2a-i? *

Copy and paste relevant sections from the manuscript (include quotes in quotation marks "like this" to indicate direct quotes from your manuscript), or elaborate on this item by providing additional information not in the ms, or briefly explain why the item is not applicable/relevant for your study

"In patients living with severe obesity (BMI ≥40 kg/m2 or BMI ≥35 kg/m2 with ≥1 major obesity-related complication), bariatric surgery in combination with behavioral interventions is a more effective option than behavioral therapies alone for long-term
weight loss (≥50% weight loss maintained for a minimum of 5 years) and remission of chronic conditions (such as type 2 diabetes, hypertension, sleep apnea, and dyslipidemia, as well as other conditions associated with increased adiposity) [1-3]."
", in order to improve weight loss and body composition and optimize health while minimizing postoperative complications, patients are recommended to actively adhere to a dietary protocol including vitamin and micronutrient intake and to engage in regular physical activity [5].
"In order to improve long-term results and follow-up after surgery, the use of telemedicine or eHealth has been proposed for delivering health care to surgical patients [12-16]. The concept consists of the delivery of health-related services and information using telecommunication technologies or digital devices. The addition of telemedicine to standard care could support patients in changing their health behaviors and thus improve clinical outcomes."

2a-ii) Scientific background, rationale: What is known about the (type of) system

Scientific background, rationale: What is known about the (type of) system that is the object of the study (be sure to discuss the use of similar systems for other conditions/diagnoses, if appropiate), motivation for the study, i.e. what are the reasons for and what is the context for this specific study, from which stakeholder viewpoint is the study performed, potential impact of findings [2]. Briefly justify the choice of the comparator.

subitem not at all important

1 ○

2 ○

3 ○

4 ◉

5 ○

essential

Effacer la sélection

Does your paper address subitem 2a-ii? *

Copy and paste relevant sections from the manuscript (include quotes in quotation marks "like this" to indicate direct quotes from your manuscript), or elaborate on this item by providing additional information not in the ms, or briefly explain why the item is not applicable/relevant for your study

"The paucity of studies examining how eHealth interventions can support patients to engage, as early as possible after their bariatric surgery, in an active and healthy lifestyle led to this investigation. Our study aimed to evaluate the effectiveness of a telemedicine intervention program including teleconsultation and telemonitoring with digital devices for the promotion of physical activity in the 6 first months following bariatric surgery."
"As observed by Creel et al [23], patients undergoing bariatric surgery face multiple challenges in engaging in regular physical activity. For most, this means moving beyond a long history of
inactivity, many failed attempts of becoming more active, orthopedic limitations, and poor exercise tolerance. Thus, despite resulting in substantial weight loss and improved self-efficacy for exercise, surgery needs to be complemented with behavioral interventions [24]. In this regard, eHealth interventions with features including self-monitoring and personalized feedback given remotely by a counselor are proven to be more effective than standard eHealth programs for the prevention and treatment of overweight and obesity in adults [25]. Moreover, the meta-ethnographic systematic review by Robinson et al [26] examining the use of digital and mobile technologies with patients undergoing elective surgery highlighted the effectiveness of empowered patient-centered strategies with content-tailored interventions in supporting postsurgery health

2b) In INTRODUCTION: Specific objectives or hypotheses

Does your paper address CONSORT subitem 2b? *

Copy and paste relevant sections from the manuscript (include quotes in quotation marks "like this" to indicate direct quotes from your manuscript), or elaborate on this item by providing additional information not in the ms, or briefly explain why the item is not applicable/relevant for your study

Our study aimed to evaluate the effectiveness of a telemedicine intervention program including teleconsultation and telemonitoring with digital devices for the promotion of physical activity in the 6 first months following bariatric surgery.

METHODS

3a) Description of trial design (such as parallel, factorial) including allocation ratio

Does your paper address CONSORT subitem 3a? *

Copy and paste relevant sections from the manuscript (include quotes in quotation marks "like this" to indicate direct quotes from your manuscript), or elaborate on this item by providing additional information not in the ms, or briefly explain why the item is not applicable/relevant for your study

"This study used a mixed methods design based on an open-label RCT. It integrated quantitative data collected by questionnaires and qualitative data through focus groups and in-depth individual interviews using a sequential "QUANT quali" temporality design (the quantitative component precedes the qualitative one, and it is predominant). This study combined triangulation (to converge results from different methods to validate interpretations) and complementarity (to reinforce, illustrate, or clarify the results of one method by those of another) [27]. Moreover, it applied an interpretative strategy for integrating the collected quantitative and qualitative data." "The randomization was computer-generated, and patients were allocated after they had signed the informed consent. To avoid patient dropouts in the control group, patients were randomized in 2 different and parallel intervention groups involving telemedicine: (1) remote physical activity coaching by phone led by a case manager with training in physical activity, diet, and behavior modification (TelePhys group) [28] or (2) remote dietary coaching by phone led by a dietitian (TeleDiet group) according to standard French guidelines for postbariatric follow-up [29]. The TeleDiet group was the control group for the TelePhys group and vice versa. At inclusion, each patient received 2 wireless telemonitoring devices: an electronic body weight scale (WebCoach POP CSI31360WH) and a pedometer band (Activi-T Band CTW41329BK) from Terraillon [30]. For both groups, the web platform tracked the parameters collected by the connected pedometer and scale, organized appointments with patients, and ensured traceability of exchanges with patients."

3b) Important changes to methods after trial commencement (such as eligibility criteria), with reasons

Does your paper address CONSORT subitem 3b? *

Copy and paste relevant sections from the manuscript (include quotes in quotation marks "like this" to indicate direct quotes from your manuscript), or elaborate on this item by providing additional information not in the ms, or briefly explain why the item is not applicable/relevant for your study

None important changes to methods were made after trial commencement

3b-i) Bug fixes, Downtimes, Content Changes

Bug fixes, Downtimes, Content Changes: ehealth systems are often dynamic systems. A description of changes to methods therefore also includes important changes made on the intervention or comparator during the trial (e.g., major bug fixes or changes in the functionality or content) (5-iii) and other “unexpected events” that may have influenced study design such as staff changes, system failures/downtimes, etc. [2].

subitem not at all important

- 1 (selected)
- 2
- 3
- 4
- 5

essential

Effacer la sélection

Does your paper address subitem 3b-i?

Copy and paste relevant sections from the manuscript (include quotes in quotation marks "like this" to indicate direct quotes from your manuscript), or elaborate on this item by providing additional information not in the ms, or briefly explain why the item is not applicable/relevant for your study

Our RCT was not concerned with bug fixes, downtimes ou content changes... Considering the technological problems with the connected devices (22% of participants stopped the study mostly because of equipment malfunction) we would have wished for.

4a) Eligibility criteria for participants

Does your paper address CONSORT subitem 4a? *

Copy and paste relevant sections from the manuscript (include quotes in quotation marks "like this" to indicate direct quotes from your manuscript), or elaborate on this item by providing additional information not in the ms, or briefly explain why the item is not applicable/relevant for your study

"Inclusion criteria were as follows: age
>18 years; preoperative weight less than 150 kg (due to the
technical weight limit of the scale); primary surgery only; no
postoperative complication between surgery and 1 month
postoperative; valid home web access with computer, tablet, or
smartphone; signed informed consent; ability to read and write
in the French language; and valid affiliation with the French
social security."

4a-i) Computer / Internet literacy

Computer / Internet literacy is often an implicit "de facto" eligibility criterion - this should be explicitly clarified.

subitem not at all important

1 ○

2 ◉

3 ○

4 ○

5 ○

essential

Effacer la sélection

Does your paper address subitem 4a-i?

Copy and paste relevant sections from the manuscript (include quotes in quotation marks "like this" to indicate direct quotes from your manuscript), or elaborate on this item by providing additional information not in the ms, or briefly explain why the item is not applicable/relevant for your study

"An inclusion criteria was to have valid home web access with computer, tablet, or smartphone; so our participants had already an internet/computer literacy or were living with people who could help them with it."

4a-ii) Open vs. closed, web-based vs. face-to-face assessments:

Open vs. closed, web-based vs. face-to-face assessments: Mention how participants were recruited (online vs. offline), e.g., from an open access website or from a clinic, and clarify if this was a purely web-based trial, or there were face-to-face components (as part of the intervention or for assessment), i.e., to what degree got the study team to know the participant. In online-only trials, clarify if participants were quasi-anonymous and whether having multiple identities was possible or whether technical or logistical measures (e.g., cookies, email confirmation, phone calls) were used to detect/prevent these.

subitem not at all important

- 1 ○
- 2 ○
- 3 ○
- 4 ○
- 5 ◉

essential

Effacer la sélection

Does your paper address subitem 4a-ii? *

Copy and paste relevant sections from the manuscript (include quotes in quotation marks "like this" to indicate direct quotes from your manuscript), or elaborate on this item by providing additional information not in the ms, or briefly explain why the item is not applicable/relevant for your study

"The randomization was computer-generated, and patients were allocated after they had signed the informed consent. To avoid patient dropouts in the control group, patients were randomized in 2 different and parallel intervention groups involving telemedicine: (1) remote physical activity coaching by phone led by a case manager with training in physical activity, diet, and behavior modification (TelePhys group) [28] or (2) remote dietary coaching by phone led by a dietitian (TeleDiet group) according to standard French guidelines for postbariatric follow-up [29]. The TeleDiet group was the control group for the TelePhys group and vice versa."

4a-iii) Information giving during recruitment

Information given during recruitment. Specify how participants were briefed for recruitment and in the informed consent procedures (e.g., publish the informed consent documentation as appendix, see also item X26), as this information may have an effect on user self-selection, user expectation and may also bias results.

subitem not at all important

- 1 ○
- 2 ○
- 3 ○
- 4 ○
- 5 ◉

essential

Effacer la sélection

Does your paper address subitem 4a-iii?

Copy and paste relevant sections from the manuscript (include quotes in quotation marks "like this" to indicate direct quotes from your manuscript), or elaborate on this item by providing additional information not in the ms, or briefly explain why the item is not applicable/relevant for your study

"Patients learned about the study at their last consultation with their surgeon before the operation. Those interested signed the informed consent and completed baseline questionnaires during the first postoperative week. Then, for each participant, telemedicine interventions began at 1 month postoperatively and lasted for 6 months."
"This study was conducted in accordance with the ethical standards of the institutional research committee and with the 1964 Helsinki Declaration and its later amendments or comparable ethical standards. This study was approved by the institutional medical ethical board Committee for the Protection of Persons of the Ile-de-France Region XI (approval number: IDRCB 2015-A01787-42/CPP 15053).
Informed consent was obtained from all individual participants included in the study."

4b) Settings and locations where the data were collected

Does your paper address CONSORT subitem 4b? *

Copy and paste relevant sections from the manuscript (include quotes in quotation marks "like this" to indicate direct quotes from your manuscript), or elaborate on this item by providing additional information not in the ms, or briefly explain why the item is not applicable/relevant for your study

"At inclusion, each patient received 2 wireless telemonitoring devices: an electronic body weight scale (WebCoach POP CSI31360WH) and a pedometer band (Activi-T Band CTW41329BK) from Terraillon [30]. For both groups, the web platform tracked the parameters collected by the connected pedometer and scale, organized appointments with patients, and ensured traceability of exchanges with patients."

4b-i) Report if outcomes were (self-)assessed through online questionnaires

Clearly report if outcomes were (self-)assessed through online questionnaires (as common in web-based trials) or otherwise.

subitem not at all important

1 ○

2 ○

3 ○

4 ○

5 ◉

essential

Effacer la sélection

Does your paper address subitem 4b-i? *

Copy and paste relevant sections from the manuscript (include quotes in quotation marks "like this" to indicate direct quotes from your manuscript), or elaborate on this item by providing additional information not in the ms, or briefly explain why the item is not applicable/relevant for your study

"For 6 months, patients in the TelePhys group received a monthly one-on-one tele-accompaniment of 30 minutes. To enable personalized coaching, patients' initial mobility diagnosis was based on the self-administered Recently Modified Physical Activity Questionnaire (RMPAQ) [31,32] and additional questions to determine the patient's barriers and motivation to using different means of locomotion (car, walking, public transport, active transport) for home-to-work travel, daily utility travel, and leisure, as well as their barriers and motivation to change to more physically active means of locomotion. In particular, the RMPAQ consists of 9 questions covering 4 areas of physical activity: domestic life, work, leisure, and transportation [31]. It includes questions about the use of a computer, television viewing, and climbing stairs at home. The travel section concerns 4 usual modes of locomotion: walking, cycling, car, and public transport."

4b-ii) Report how institutional affiliations are displayed

Report how institutional affiliations are displayed to potential participants [on ehealth media], as affiliations with prestigious hospitals or universities may affect volunteer rates, use, and reactions with regards to an intervention.(Not a required item – describe only if this may bias results)

subitem not at all important

1 (selected)

2

3

4

5

essential

Effacer la sélection

Does your paper address subitem 4b-ii?

Copy and paste relevant sections from the manuscript (include quotes in quotation marks "like this" to indicate direct quotes from your manuscript), or elaborate on this item by providing additional information not in the ms, or briefly explain why the item is not applicable/relevant for your study

Not concerned as participants were patients who underwent bariatric surgery (gastric bypass, sleeve, or adjustable gastric band) in 2 university teaching public hospitals in the Paris area (France)

5) The interventions for each group with sufficient details to allow replication, including how and when they were actually administered

5-i) Mention names, credential, affiliations of the developers, sponsors, and owners

Mention names, credential, affiliations of the developers, sponsors, and owners [6] (if authors/evaluators are owners or developer of the software, this needs to be declared in a “Conflict of interest” section or mentioned elsewhere in the manuscript).

subitem not at all important

1 (selected)

2

3

4

5

essential

Effacer la sélection

Does your paper address subitem 5-i?

Copy and paste relevant sections from the manuscript (include quotes in quotation marks "like this" to indicate direct quotes from your manuscript), or elaborate on this item by providing additional information not in the ms, or briefly explain why the item is not applicable/relevant for your study

We combined already existant digital devices (connected wireless pedometer and scale : (WebCoach POP CSI31360WH) and (Activi-T Band CTW41329BK) from Terraillon) and telemedicine (on physical activity or diet counseling) to strengthen patients’ empowerment to play an active role in their long-term care after bariatric surgery. Interventions combined education with behavioral patient-centered strategies and were
delivered in a collaborative way that respected patient autonomy. Indications to perform surgeries we followed relied on the guidelines published by the National Institute of HealthHAS 2009 Obésité : prise en charge chirurgicale chez l'adulte RECOMMANDATION DE BONNE PRATIQUE du 22 juin 2009

5-ii) Describe the history/development process

Describe the history/development process of the application and previous formative evaluations (e.g., focus groups, usability testing), as these will have an impact on adoption/use rates and help with interpreting results.

subitem not at all important

1 ○

2 ○

3 ○

4 ○

5 ○

essential

Does your paper address subitem 5-ii?

Copy and paste relevant sections from the manuscript (include quotes in quotation marks "like this" to indicate direct quotes from your manuscript), or elaborate on this item by providing additional information not in the ms, or briefly explain why the item is not applicable/relevant for your study

It is accuratedly described in our paper

5-iii) Revisions and updating

Revisions and updating. Clearly mention the date and/or version number of the application/intervention (and comparator, if applicable) evaluated, or describe whether the intervention underwent major changes during the evaluation process, or whether the development and/or content was “frozen” during the trial. Describe dynamic components such as news feeds or changing content which may have an impact on the replicability of the intervention (for unexpected events see item 3b).

subitem not at all important

- 1 ○
- 2 ◉
- 3 ○
- 4 ○
- 5 ○

essential

Effacer la sélection

Does your paper address subitem 5-iii?

Copy and paste relevant sections from the manuscript (include quotes in quotation marks "like this" to indicate direct quotes from your manuscript), or elaborate on this item by providing additional information not in the ms, or briefly explain why the item is not applicable/relevant for your study

There were no revisions and updating of the apps used

5-iv) Quality assurance methods

Provide information on quality assurance methods to ensure accuracy and quality of information provided [1], if applicable.

subitem not at all important

1 ○

2 ◉

3 ○

4 ○

5 ○

essential

Effacer la sélection

Does your paper address subitem 5-iv?

Copy and paste relevant sections from the manuscript (include quotes in quotation marks "like this" to indicate direct quotes from your manuscript), or elaborate on this item by providing additional information not in the ms, or briefly explain why the item is not applicable/relevant for your study

"This study was conducted in accordance with the ethical standards of the institutional research committee and with the 1964 Helsinki Declaration and its later amendments or comparable ethical standards. This study was approved by the institutional medical ethical board Committee for the Protection of Persons of the Ile-de-France Region XI (approval number: IDRCB 2015-A01787-42/CPP 15053)."

5-v) Ensure replicability by publishing the source code, and/or providing screenshots/screen-capture video, and/or providing flowcharts of the algorithms used

Ensure replicability by publishing the source code, and/or providing screenshots/screen-capture video, and/or providing flowcharts of the algorithms used. Replicability (i.e., other researchers should in principle be able to replicate the study) is a hallmark of scientific reporting.

subitem not at all important

1 ○

2 ◉

3 ○

4 ○

5 ○

essential

Effacer la sélection

Does your paper address subitem 5-v?

Copy and paste relevant sections from the manuscript (include quotes in quotation marks "like this" to indicate direct quotes from your manuscript), or elaborate on this item by providing additional information not in the ms, or briefly explain why the item is not applicable/relevant for your study

We did not included this option in our informed consent letter. So, it is not allowed by the institutional medical ethical board Committee for the Protection
of Persons of the Ile-de-France Region XI (approval number:
IDRCB 2015-A01787-42/CPP 15053)."

5-vi) Digital preservation

Digital preservation: Provide the URL of the application, but as the intervention is likely to change or disappear over the course of the years; also make sure the intervention is archived (Internet Archive, webcitation.org, and/or publishing the source code or screenshots/videos alongside the article). As pages behind login screens cannot be archived, consider creating demo pages which are accessible without login.

subitem not at all important

1 (selected)

2

3

4

5

essential

Effacer la sélection

Does your paper address subitem 5-vi?

Copy and paste relevant sections from the manuscript (include quotes in quotation marks "like this" to indicate direct quotes from your manuscript), or elaborate on this item by providing additional information not in the ms, or briefly explain why the item is not applicable/relevant for your study

We did not included this option in our informed consent letter. So, it is not allowed by the institutional medical ethical board Committee for the Protection
of Persons of the Ile-de-France Region XI (approval number:
IDRCB 2015-A01787-42/CPP 15053)."

5-vii) Access

Access: Describe how participants accessed the application, in what setting/context, if they had to pay (or were paid) or not, whether they had to be a member of specific group. If known, describe how participants obtained “access to the platform and Internet” [1]. To ensure access for editors/reviewers/readers, consider to provide a “backdoor” login account or demo mode for reviewers/readers to explore the application (also important for archiving purposes, see vi).

subitem not at all important

- 1 (selected)
- 2
- 3
- 4
- 5

essential

Effacer la sélection

Does your paper address subitem 5-vii? *

Copy and paste relevant sections from the manuscript (include quotes in quotation marks "like this" to indicate direct quotes from your manuscript), or elaborate on this item by providing additional information not in the ms, or briefly explain why the item is not applicable/relevant for your study

The apps used belong to Teraillon, not to us. PArticipants were not payed to use them.

5-viii) Mode of delivery, features/functionalities/components of the intervention and comparator, and the theoretical framework

Describe mode of delivery, features/functionalities/components of the intervention and comparator, and the theoretical framework [6] used to design them (instructional strategy [1], behaviour change techniques, persuasive features, etc., see e.g., [7, 8] for terminology). This includes an in-depth description of the content (including where it is coming from and who developed it) [1]," whether [and how] it is tailored to individual circumstances and allows users to track their progress and receive feedback" [6]. This also includes a description of communication delivery channels and – if computer-mediated communication is a component – whether communication was synchronous or asynchronous [6]. It also includes information on presentation strategies [1], including page design principles, average amount of text on pages, presence of hyperlinks to other resources, etc. [1].

subitem not at all important

1 ○

2 ○

3 ◉

4 ○

5 ○

essential

Effacer la sélection

Does your paper address subitem 5-viii? *

Copy and paste relevant sections from the manuscript (include quotes in quotation marks "like this" to indicate direct quotes from your manuscript), or elaborate on this item by providing additional information not in the ms, or briefly explain why the item is not applicable/relevant for your study

"At inclusion, each patient
received 2 wireless telemonitoring devices: an electronic body
weight scale (WebCoach POP CSI31360WH) and a pedometer
band (Activi-T Band CTW41329BK) from Terraillon"

5-ix) Describe use parameters

Describe use parameters (e.g., intended “doses” and optimal timing for use). Clarify what instructions or recommendations were given to the user, e.g., regarding timing, frequency, heaviness of use, if any, or was the intervention used ad libitum.

subitem not at all important

1 ○

2 ○

3 ○

4 ◉

5 ○

essential

Effacer la sélection

Does your paper address subitem 5-ix?

Copy and paste relevant sections from the manuscript (include quotes in quotation marks "like this" to indicate direct quotes from your manuscript), or elaborate on this item by providing additional information not in the ms, or briefly explain why the item is not applicable/relevant for your study

"The web platform tracked the parameters collected by the connected pedometer and scale, organized appointments with patients, and ensured traceability of exchanges with patients"

5-x) Clarify the level of human involvement

Clarify the level of human involvement (care providers or health professionals, also technical assistance) in the e-intervention or as co-intervention (detail number and expertise of professionals involved, if any, as well as “type of assistance offered, the timing and frequency of the support, how it is initiated, and the medium by which the assistance is delivered”. It may be necessary to distinguish between the level of human involvement required for the trial, and the level of human involvement required for a routine application outside of a RCT setting (discuss under item 21 – generalizability).

subitem not at all important

1 ○

2 ○

3 ○

4 ○

5 ◉

essential

Effacer la sélection

Does your paper address subitem 5-x?

Copy and paste relevant sections from the manuscript (include quotes in quotation marks "like this" to indicate direct quotes from your manuscript), or elaborate on this item by providing additional information not in the ms, or briefly explain why the item is not applicable/relevant for your study

Level of human involvement quite high from care providers and health professionals : "For 6 months, patients in the TelePhys group received a monthly one-on-one tele-accompaniment of 30 minutes. To enable personalized coaching, patients' initial mobility diagnosis was based on the self-administered Recently Modified Physical Activity Questionnaire (RMPAQ) [31,32] and additional questions to determine the patient's barriers and motivation to using different means of locomotion (car, walking, public transport, active transport) for home-to-work travel, daily utility travel, and leisure, as well as their barriers and motivation to change to more physically active means of locomotion. In particular, the RMPAQ consists of 9 questions covering 4 areas of physical activity: domestic life, work, leisure, and transportation [31]. It includes questions about the use of a computer, television viewing, and climbing stairs at home. The travel section concerns 4 usual modes of locomotion: walking, cycling, car, and public transport. The TelePhys coach acted as

a case manager who followed the patient's return to a more active lifestyle (engaging in a sport, doing regular exercise at home, dynamic walking or gardening, use of daily active modes of locomotion) in order to keep them motivated in their efforts, individually guide them in their daily travel choices, and help them solve problems. The TelePhys intervention was not based on a general outcome goal (such as reaching 10,000 steps per day or 150 minutes of structured exercise per week) that was communicated to patients. Instead, it was process-oriented, focusing on empowering and educating patients on self-monitoring while collaboratively setting personalized goals that reflected their aims, values, and current condition within the context of their environment (available equipment, resources, and social support).
The TeleDiet intervention also consisted of one-on-one coaching teleconsultation that lasted 30 minutes and was repeated monthly for 6 months. The dietitian who conducted them followed each patient's diet and eating practices in order to provide them personalized advice on how to adjust to the postoperative dietary recommendations (namely, to multiply the daily meals, each being of a reduced volume; chew food well; limit drinks during meals; and avoid soft drinks)."

5-xi) Report any prompts/reminders used

Report any prompts/reminders used: Clarify if there were prompts (letters, emails, phone calls, SMS) to use the application, what triggered them, frequency etc. It may be necessary to distinguish between the level of prompts/reminders required for the trial, and the level of prompts/reminders for a routine application outside of a RCT setting (discuss under item 21 – generalizability).

subitem not at all important

1 ○

2 ○

3 ○

4 ○

5 ◉

essential

Effacer la sélection

Does your paper address subitem 5-xi? *

Copy and paste relevant sections from the manuscript (include quotes in quotation marks "like this" to indicate direct quotes from your manuscript), or elaborate on this item by providing additional information not in the ms, or briefly explain why the item is not applicable/relevant for your study

YEs : "For 6 months, patients in the TelePhys group received a monthly one-on-one tele-accompaniment of 30 minutes. "

"The TeleDiet intervention also consisted of one-on-one coaching teleconsultation that lasted 30 minutes and was repeated monthly for 6 months."

5-xii) Describe any co-interventions (incl. training/support)

Describe any co-interventions (incl. training/support): Clearly state any interventions that are provided in addition to the targeted eHealth intervention, as ehealth intervention may not be designed as stand-alone intervention. This includes training sessions and support [1]. It may be necessary to distinguish between the level of training required for the trial, and the level of training for a routine application outside of a RCT setting (discuss under item 21 – generalizability.

subitem not at all important

1 ○

2 ○

3 ○

4 ○

5 ◉

essential

Effacer la sélection

Does your paper address subitem 5-xii? *

Copy and paste relevant sections from the manuscript (include quotes in quotation marks "like this" to indicate direct quotes from your manuscript), or elaborate on this item by providing additional information not in the ms, or briefly explain why the item is not applicable/relevant for your study

co-intervention is accurately detailed in the paper : it combined digital devices (connected wireless pedometer and scale) and telemedicine (on physical activity or diet counseling) to strengthen patients' empowerment to play an active role in their long-term care after bariatric surgery. Interventions combined education with behavioral patient-centered strategies and were delivered in a collaborative way that respected patient autonomy. This RCT used a complementary qualitative approach to reinforce, illustrate, or clarify results.

6a) Completely defined pre-specified primary and secondary outcome measures, including how and when they were assessed

Does your paper address CONSORT subitem 6a? *

Copy and paste relevant sections from the manuscript (include quotes in quotation marks "like this" to indicate direct quotes from your manuscript), or elaborate on this item by providing additional information not in the ms, or briefly explain why the item is not applicable/relevant for your study

"The primary outcome of this study was the difference in the mean number of steps measured during a period of 14 days at the first and sixth postoperative months between the TelePhys and TeleDiet groups. Data were collected using the connected wireless watch pedometer. Weight change was analyzed during the 6 postoperative months using the connected weight scale and reported as weight in kilograms and percent weight loss.
A series of focus groups with 4 to 6 participants each was conducted at the end of patients' participation in the TelePhys or TeleDiet intervention. For the patients who were not available or did not feel comfortable with speaking in a group, the option of an individual interview was given. Focus groups and interviews aimed to qualitatively capture the patients' experiences with the telemedicine provided, their use of the connected watch pedometer and body weight scale, and the effects of the intervention on their lifestyle after bariatric surgery."

6a-i) Online questionnaires: describe if they were validated for online use and apply CHERRIES items to describe how the questionnaires were designed/deployed

If outcomes were obtained through online questionnaires, describe if they were validated for online use and apply CHERRIES items to describe how the questionnaires were designed/deployed [9].

subitem not at all important

- 1 (selected)
- 2
- 3
- 4
- 5

essential

Effacer la sélection

Does your paper address subitem 6a-i?

Copy and paste relevant sections from manuscript text

We used the self-administered Recently Modified Physical Activity Questionnaire (RMPAQ) completed with additional questions to determine the patient's barriers and motivation to using different means of locomotion (car, walking, public transport, active transport) for home-to-work travel, daily utility travel, and leisure, as well as their barriers and motivation to change to more physically active means of locomotion.

6a-ii) Describe whether and how "use" (including intensity of use/dosage) was defined/measured/monitored

Describe whether and how "use" (including intensity of use/dosage) was defined/measured/monitored (logins, logfile analysis, etc.). Use/adoption metrics are important process outcomes that should be reported in any ehealth trial.

subitem not at all important

1 ○

2 ○

3 ○

4 ○

5 ◉

essential

Effacer la sélection

Does your paper address subitem 6a-ii?

Copy and paste relevant sections from manuscript text

Use metrics were important process outcomes for our telemedecine trial; They are a main part of our results

6a-iii) Describe whether, how, and when qualitative feedback from participants was obtained

Describe whether, how, and when qualitative feedback from participants was obtained (e.g., through emails, feedback forms, interviews, focus groups).

subitem not at all important

1 ○

2 ○

3 ○

4 ○

5 ◉

essential

Effacer la sélection

Does your paper address subitem 6a-iii?

Copy and paste relevant sections from manuscript text

"Due to research budget restrictions, the qualitative part of the study could only be proposed to the first one-half of patients who completed the 6-month intervention (n=35). A total of 18 participants agreed to be interviewed (8 patients in the TelePhys group and 10 patients in the TeleDiet group); 3 focus groups of 4 participants each and 6 individual interviews were finally conducted. Among the TelePhys patients who participated in the qualitative part of the study, one-half opted for an individual interview (4/8, 50%), and the other one-half opted for the focus group (4/8, 50%). Among the 10 TeleDiet patients, 2 chose the individual interview, and 8 participated in a focus group.
For all these participants, “physical activity” was first associated with “sports,” defined as intense, tiring exercises that are practiced in leisure time, with the guidance of a sports instructor.
When we are told “you need to do more physical activity,” I directly think about going to the gym. [P6, TelePhys, interview]
For me, it’s getting back into physical condition, at the level of endurance and breathing again, a kind of little punch. All the things I didn’t have before. (...) So, I started to go to the fitness room 3 weeks ago. [P12, TeleDiet, interview]
Patients in the TelePhys group also mentioned day-to-day activities requiring bodily efforts such as “household,” “gardening,” “taking the stairs instead of the lift,” and “preferring walking to the bus,” actions that they understand better as “mobility activities.”
Now I know that everything that is daily activity (cleaning etc) is considered physical activity, but this is not the first thing I think about as “physical activity” but as “mobility,” namely all that can increase my physical activity in everyday life: to prefer walking to the bus, stairs to elevators, in order to try to move more. [P1, TelePhys, interview]
If sport was viewed as producing suffering and discomfort, mobility practices such as walking or biking for their home or work travels were seen as a source of mental wellness.
Physical activity? It’s gym, pain, sweat [laughter shared by all], stuff that hurts, that doesn’t make you want to. [P5, TelePhys, focus group]
When I go home, I’m in my head. I have my music. I want to think about something else, to evacuate, so I get off the bus some stops earlier. It’s true; it feels good. [P4, TelePhys, interview]
I have just moved to a city that is more suitable for bicycle transport (...), and it feels so good going back home by bike. [P6, Telephys, focus group]
Despite their weight loss and willingness, participants expressed difficulties in integrating higher and sustained physical activity in the very context of their day-to-day lives. The 3 factors most commonly mentioned as jeopardizing their efforts to decrease their sedentary lifestyle were (1) professional constraints, in

their sedentary lifestyle were (1) professional constraints, in particular extended working hours and sedentary office work; (2) the long Paris-suburban areas of home-to-work travel time; and (3) lack of urban policies that would promote outdoor activities with either practicable green areas, safe pedestrian walkways, and bike path network and investment in sport infrastructure that is accessible by everyone.

I'm working at a bank (...) in a high responsibility working position. There are weeks of hyperintensive work. These last few weeks, I have been going to work at 7 AM and coming home at 11 PM (...) This weekend, I also stayed for long hours working on the computer. [P17, TeleDiet, interview]

From home to my office, I put in 1 hour 20 minutes. Public transport is so direct that I barely walk. Of course, I could try to get off earlier and continue on foot, but it is really my schedule that...because I leave very early from home and come back late. Also, to be honest, my neighborhood doesn't make you want to walk more, especially when it gets dark. [P4, TelePhys, focus group]

I tried cycling in Paris. It was one of my goals to lose weight. But, take the bike paths: There aren't many, they're on the sidewalk, so they're overrun by pedestrians. It's a 2-way street, so people sometimes go from right to left. Then, the bus corridors: It's not bad, but on the security level, it's still to be seen. Buses are aware, pay attention, but taxis that use the bus tracks are not. Well, too stressful, a real danger. [P7, TelePhys, focus group]

In our neighborhood, parks are not clean. There are drunk people or taking drugs or whatever, so not a very pleasant environment for a promenade. [P8, TelePhys, focus group]

Self-presentational concerns were also mentioned by a few patients, who also expressed how establishing new routines takes time.

I love dancing, but I really feel the need to lose more weight to take dance classes. Well, it's in my head, I know. I can't avoid thinking that it's maybe more beautiful to see someone slimmer who dances than someone round shaped. [Interviewer: Where are you now on this project?] Maybe, uh, a little more concrete. I visualize it a bit better, but maybe I'll wait a little longer for me to feel better. [P2, TelePhys, interview]

The primary facilitator for engaging in a sport activity was weight loss, not only because it decreased pain and physical strain but also because it was felt as a response to the fatigue brought by rapid and intensive body mass loss.

I felt quite weak because of my weight loss, and I still feel it now. So, I started to go to the gym to regain a little of strength and energy. [P12, TeleDiet, focus

6b) Any changes to trial outcomes after the trial commenced, with reasons

Does your paper address CONSORT subitem 6b? *

Copy and paste relevant sections from the manuscript (include quotes in quotation marks "like this" to indicate direct quotes from your manuscript), or elaborate on this item by providing additional information not in the ms, or briefly explain why the item is not applicable/relevant for your study

No changes were observed as regards "trial outcomes after the trial commenced

7a) How sample size was determined

NPT: When applicable, details of whether and how the clustering by care provides or centers was addressed

7a-i) Describe whether and how expected attrition was taken into account when calculating the sample size

Describe whether and how expected attrition was taken into account when calculating the sample size

subitem not at all important

1 ○
2 ○
3 ○
4 ○
5 ●

essential

Effacer la sélection

group]
Close friends, family members, and work colleagues were also highlighted as important support in their lifestyle change, especially in making them discover new sports.
I started cycling with my daughters and wife on weekends in the countryside, next to Paris. [P5, TelePhys, interview]
I do aquagym on Thursday evenings. A cousin was attending the course, and I joined her. [P1, TelePhys, interview]
Near my office, there is a sports club doing things in the evenings. (...) I have a coworker who was doing aquagym there, and now we go together. [P9, TeleDiet, focus group]
In general, patients considered digital health devices as useful tools to help them start implementing postoperative prescriptions for physical activity. Self-tracking of their steps, weight loss, or fat-muscle rebalancing in the present time was highlighted as a method that allowed them to objectify their physical changes. Digital devices brought them well-defined benchmarks to inform them on their efforts on a daily basis.
It is very encouraging when you see the curves of weight loss and of the changes in the body mass composition, what has changed inside of your body in terms of fat, water, muscle, etc. [P16, TeleDiet, focus group]
We know that we're losing weight and body mass, but to be able to see the fat loss or muscle gain, I find it very nice. It motivated me to go on. [P1, TelePhys, interview]
I used to check the watch pedometer more at the end of the day when coming back home: "I did that many steps so far"; it gave me an indicator, "Okay, I'll walk a little more." So, I'll get off at a few stations before with the idea of increasing the number of steps to reach at least my daily goal of 10,000 steps. [P5, TelePhys, focus group]
TelePhys patients considered the teleconsultations as a helpful complementary tool for their postsurgery follow-up to strengthen their adherence to postoperative recommendations. We drew 3 major reasons from their feedback: (1) It boosted their motivation; (2) it gave specific information that reassured them regarding their intense, surgery-driven body changes; and (3) it provided tailored advice according to their socioeconomic situation and changing physical and psychological needs.
The regular contact with the coach helped me to keep motivated to change my eating habits and start moving more. [P1, TelePhys, interview]
Having an expert with whom to speak regularly by phone about my situation was reassuring. (...) Even if the medical team had previously informed us about

Does your paper address subitem 7a-i?

Copy and paste relevant sections from manuscript title (include quotes in quotation marks "like this" to indicate direct quotes from your manuscript), or elaborate on this item by providing additional information not in the ms, or briefly explain why the item is not applicable/relevant for your study

As this study is the first multicentric RCT that combined digital devices (connected wireless pedometer and scale) and telemedicine (on physical activity or diet counseling) to strengthen patients' empowerment to play an active role in their long-term care after bariatric surgery as well as for financial and time constraints we recruited 90 patients from June 2016 to July 2018, and data collection was completed in February 2019.

7b) When applicable, explanation of any interim analyses and stopping guidelines

Does your paper address CONSORT subitem 7b? *

Copy and paste relevant sections from the manuscript (include quotes in quotation marks "like this" to indicate direct quotes from your manuscript), or elaborate on this item by providing additional information not in the ms, or briefly explain why the item is not applicable/relevant for your study

We did consider this within our research team; this is included in the protocol study we submited to the ethnical research committee.

8a) Method used to generate the random allocation sequence

NPT: When applicable, how care providers were allocated to each trial group

the weight loss process and food changes after the surgery, these are actually hard to go through once back home. [P4, TelePhys, interview]
The coach asked me what I was doing in my real life so we could work together on how to customize. [P7, TelePhys, focus group]
To have someone who is there and who really adapts things to our case and who does not give courses that are a little more general. [P2, TelePhys, interview]
It gave immediate response to the problems and difficulties I was going through to be more active. [P5, TelePhys, focus group]"

Does your paper address CONSORT subitem 8a? *

Copy and paste relevant sections from the manuscript (include quotes in quotation marks "like this" to indicate direct quotes from your manuscript), or elaborate on this item by providing additional information not in the ms, or briefly explain why the item is not applicable/relevant for your study

According to their competences : "remote physical activity coaching by phone was led by a case manager with training in physical activity, diet, and behavior modification (TelePhys group)" and "remote dietary coaching by phone led by a dietitian (TeleDiet group) according to standard French guidelines for postbariatric follow-up"

8b) Type of randomisation; details of any restriction (such as blocking and block size)

Does your paper address CONSORT subitem 8b? *

Copy and paste relevant sections from the manuscript (include quotes in quotation marks "like this" to indicate direct quotes from your manuscript), or elaborate on this item by providing additional information not in the ms, or briefly explain why the item is not applicable/relevant for your study

"The randomization was computer-generated, and patients were allocated after they had signed the informed consent. To avoid patient dropouts in the control group, patients were randomized in 2 different and parallel intervention groups involving telemedicine: (1) remote physical activity coaching by phone led by a case manager with training in physical activity, diet, and behavior modification (TelePhys group) [28] or (2) remote dietary coaching by phone led by a dietitian (TeleDiet group) according to standard French guidelines for postbariatric follow-up [29]. The TeleDiet group was the control group for the TelePhys group and vice versa"

9) Mechanism used to implement the random allocation sequence (such as sequentially numbered containers), describing any steps taken to conceal the sequence until interventions were assigned

Does your paper address CONSORT subitem 9? *

Copy and paste relevant sections from the manuscript (include quotes in quotation marks "like this" to indicate direct quotes from your manuscript), or elaborate on this item by providing additional information not in the ms, or briefly explain why the item is not applicable/relevant for your study

The randomization was computer-generated, and patients were allocated after they had signed the informed consent. To avoid patient dropouts in the control group, patients were randomized in 2 different and parallel intervention groups involving telemedicine: (1) remote physical activity coaching by phone led by a case manager with training in physical activity, diet, and behavior modification (TelePhys group) [28] or (2) remote dietary coaching by phone led by a dietitian (TeleDiet group) according to standard French guidelines for postbariatric follow-up [29]. The TeleDiet group was the control group for the TelePhys group and vice versa

10) Who generated the random allocation sequence, who enrolled participants, and who assigned participants to interventions

Does your paper address CONSORT subitem 10? *

Copy and paste relevant sections from the manuscript (include quotes in quotation marks "like this" to indicate direct quotes from your manuscript), or elaborate on this item by providing additional information not in the ms, or briefly explain why the item is not applicable/relevant for your study

The randomization was computer-generated, and patients were allocated after they had signed the informed consent. To avoid patient dropouts in the control group, patients were randomized in 2 different and parallel intervention groups involving telemedicine: (1) remote physical activity coaching by phone led by a case manager with training in physical activity, diet, and behavior modification (TelePhys group) [28] or (2) remote dietary coaching by phone led by a dietitian (TeleDiet group) according to standard French guidelines for postbariatric follow-up [29]. The TeleDiet group was the control group for the TelePhys group and vice versa

11a) If done, who was blinded after assignment to interventions (for example, participants, care providers, those assessing outcomes) and how

NPT: Whether or not administering co-interventions were blinded to group assignment

11a-i) Specify who was blinded, and who wasn't

Specify who was blinded, and who wasn't. Usually, in web-based trials it is not possible to blind the participants [1, 3] (this should be clearly acknowledged), but it may be possible to blind outcome assessors, those doing data analysis or those administering co-interventions (if any).

subitem not at all important

- 1 (selected)
- 2
- 3
- 4
- 5

essential

Effacer la sélection

Does your paper address subitem 11a-i? *

Copy and paste relevant sections from the manuscript (include quotes in quotation marks "like this" to indicate direct quotes from your manuscript), or elaborate on this item by providing additional information not in the ms, or briefly explain why the item is not applicable/relevant for your study

Our RCT was an open-labelled. No blinded participants or professionals after assignment to interventions

11a-ii) Discuss e.g., whether participants knew which intervention was the “intervention of interest” and which one was the “comparator”

Informed consent procedures (4a-ii) can create biases and certain expectations - discuss e.g., whether participants knew which intervention was the “intervention of interest” and which one was the “comparator”.

subitem not at all important

- 1 (selected)
- 2
- 3
- 4
- 5

essential

Effacer la sélection

Does your paper address subitem 11a-ii?

Copy and paste relevant sections from the manuscript (include quotes in quotation marks "like this" to indicate direct quotes from your manuscript), or elaborate on this item by providing additional information not in the ms, or briefly explain why the item is not applicable/relevant for your study

For ethical issues, we did not proceed as such. In our research study, The TeleDiet group was the control group for the TelePhys group and vice versa

11b) If relevant, description of the similarity of interventions

(this item is usually not relevant for ehealth trials as it refers to similarity of a placebo or sham intervention to a active medication/intervention)

Does your paper address CONSORT subitem 11b? *

Copy and paste relevant sections from the manuscript (include quotes in quotation marks "like this" to indicate direct quotes from your manuscript), or elaborate on this item by providing additional information not in the ms, or briefly explain why the item is not applicable/relevant for your study

This is not relevant here

12a) Statistical methods used to compare groups for primary and secondary outcomes

NPT: When applicable, details of whether and how the clustering by care providers or centers was addressed

Does your paper address CONSORT subitem 12a? *

Copy and paste relevant sections from the manuscript (include quotes in quotation marks "like this" to indicate direct quotes from your manuscript), or elaborate on this item by providing additional information not in the ms, or briefly explain why the item is not applicable/relevant for your study

"Statistical results were reported according to the criteria in the CONSORT statement. Intention-to-treat analysis was performed. Quantitative and qualitative variables were described by means (SDs) and frequencies (%). We tested for potential differences using analysis of variance or Kruskall-Wallis tests for continuous variables and Fisher exact tests for discrete variables. Paired t tests were used for intragroup comparisons. Data analyses were conducted using SAS version 9.4 (SAS Institute), and P<.05 was considered to indicate statistical significance."

12a-i) Imputation techniques to deal with attrition / missing values

Imputation techniques to deal with attrition / missing values: Not all participants will use the intervention/comparator as intended and attrition is typically high in ehealth trials. Specify how participants who did not use the application or dropped out from the trial were treated in the statistical analysis (a complete case analysis is strongly discouraged, and simple imputation techniques such as LOCF may also be problematic [4]).

subitem not at all important

1 (selected)

2

3

4

5

essential

Effacer la sélection

Does your paper address subitem 12a-i? *

Copy and paste relevant sections from the manuscript (include quotes in quotation marks "like this" to indicate direct quotes from your manuscript), or elaborate on this item by providing additional information not in the ms, or briefly explain why the item is not applicable/relevant for your study

We didn’t do any replacement of missing data.

12b) Methods for additional analyses, such as subgroup analyses and adjusted analyses

Does your paper address CONSORT subitem 12b? *

Copy and paste relevant sections from the manuscript (include quotes in quotation marks "like this" to indicate direct quotes from your manuscript), or elaborate on this item by providing additional information not in the ms, or briefly explain why the item is not applicable/relevant for your study

We didn’t do any additional analyses yet

X26) REB/IRB Approval and Ethical Considerations [recommended as subheading under "Methods"] (not a CONSORT item)

X26-i) Comment on ethics committee approval

subitem not at all important

1 ○

2 ○

3 ○

4 ○

5 ◉

essential

Effacer la sélection

Does your paper address subitem X26-i?

Copy and paste relevant sections from the manuscript (include quotes in quotation marks "like this" to indicate direct quotes from your manuscript), or elaborate on this item by providing additional information not in the ms, or briefly explain why the item is not applicable/relevant for your study

"This study was conducted in accordance with the ethical standards of the institutional research committee and with the 1964 Helsinki Declaration and its later amendments or comparable ethical standards. This study was approved by the institutional medical ethical board Committee for the Protection of Persons of the Ile-de-France Region XI (approval number: IDRCB 2015-A01787-42/CPP 15053)."

x26-ii) Outline informed consent procedures

Outline informed consent procedures e.g., if consent was obtained offline or online (how? Checkbox, etc.?), and what information was provided (see 4a-ii). See [6] for some items to be included in informed consent documents.

subitem not at all important

- 1 ○
- 2 ○
- 3 ○
- 4 ○
- 5 ◉

essential

Effacer la sélection

Does your paper address subitem X26-ii?

Copy and paste relevant sections from the manuscript (include quotes in quotation marks "like this" to indicate direct quotes from your manuscript), or elaborate on this item by providing additional information not in the ms, or briefly explain why the item is not applicable/relevant for your study

"Informed consent was obtained from all individual participants
included in the study." "The randomization was computer-generated, and patients were allocated after they had signed the informed consent" "Patients learned about the study at their last consultation with their surgeon before the operation. Those interested signed the informed consent and completed baseline questionnaires during the first postoperative week. Then, for each participant, telemedicine interventions began at 1 month postoperatively and lasted for 6 months." Inclusion criteria included signed informed consent.

X26-iii) Safety and security procedures

Safety and security procedures, incl. privacy considerations, and any steps taken to reduce the likelihood or detection of harm (e.g., education and training, availability of a hotline)

subitem not at all important

1 ○

2 ○

3 ○

4 ○

5 ◉

essential

Effacer la sélection

Does your paper address subitem X26-iii?

Copy and paste relevant sections from the manuscript (include quotes in quotation marks "like this" to indicate direct quotes from your manuscript), or elaborate on this item by providing additional information not in the ms, or briefly explain why the item is not applicable/relevant for your study

Inform consent letter includes adresses and telephone number of PI and CNIL. Public Hospitals in France make available users' right services for patients to complaint. CNIL in France controls all safety and security research procedures. Ethical commitees and CNIL authorised our research protocol.

RESULTS

13a) For each group, the numbers of participants who were randomly assigned, received intended treatment, and were analysed for the primary outcome

NPT: The number of care providers or centers performing the intervention in each group and the number of patients treated by each care provider in each center

Does your paper address CONSORT subitem 13a? *

Copy and paste relevant sections from the manuscript (include quotes in quotation marks "like this" to indicate direct quotes from your manuscript), or elaborate on this item by providing additional information not in the ms, or briefly explain why the item is not applicable/relevant for your study

2 care providers and 2 public hospitals recruted participants for each group. Researchers from the Method team did the randomnisation as we have already described it.

13b) For each group, losses and exclusions after randomisation, together with reasons

Does your paper address CONSORT subitem 13b? (NOTE: Preferably, this is shown in a CONSORT flow diagram) *

Copy and paste relevant sections from the manuscript (include quotes in quotation marks "like this" to indicate direct quotes from your manuscript), or elaborate on this item by providing additional information not in the ms, or briefly explain why the item is not applicable/relevant for your study

It is explicited in the paper

13b-i) Attrition diagram

Strongly recommended: An attrition diagram (e.g., proportion of participants still logging in or using the intervention/comparator in each group plotted over time, similar to a survival curve) or other figures or tables demonstrating usage/dose/engagement.

subitem not at all important

1 ○

2 ○

3 ○

4 ○

5 ◉

essential

Effacer la sélection

Does your paper address subitem 13b-i?

Copy and paste relevant sections from the manuscript or cite the figure number if applicable (include quotes in quotation marks "like this" to indicate direct quotes from your manuscript), or elaborate on this item by providing additional information not in the ms, or briefly explain why the item is not applicable/relevant for your study

We have included an attrition diagram

14a) Dates defining the periods of recruitment and follow-up

Does your paper address CONSORT subitem 14a? *

Copy and paste relevant sections from the manuscript (include quotes in quotation marks "like this" to indicate direct quotes from your manuscript), or elaborate on this item by providing additional information not in the ms, or briefly explain why the item is not applicable/relevant for your study

We have included dates defining periods of recruitment and end of the data collection.

14a-i) Indicate if critical “secular events” fell into the study period

Indicate if critical “secular events” fell into the study period, e.g., significant changes in Internet resources available or “changes in computer hardware or Internet delivery resources”

subitem not at all important

- 1 (selected)
- 2
- 3
- 4
- 5

essential

Effacer la sélection

Does your paper address subitem 14a-i?

Copy and paste relevant sections from the manuscript (include quotes in quotation marks "like this" to indicate direct quotes from your manuscript), or elaborate on this item by providing additional information not in the ms, or briefly explain why the item is not applicable/relevant for your study

No critical secular events other than patient drop were observed

14b) Why the trial ended or was stopped (early)

Does your paper address CONSORT subitem 14b? *

Copy and paste relevant sections from the manuscript (include quotes in quotation marks "like this" to indicate direct quotes from your manuscript), or elaborate on this item by providing additional information not in the ms, or briefly explain why the item is not applicable/relevant for your study

We wanted to observed a specific time period: 6 first months after bariatric surgery For Financial, time constraints we could not extend this.

15) A table showing baseline demographic and clinical characteristics for each group

NPT: When applicable, a description of care providers (case volume, qualification, expertise, etc.) and centers (volume) in each group

Does your paper address CONSORT subitem 15? *

Copy and paste relevant sections from the manuscript (include quotes in quotation marks "like this" to indicate direct quotes from your manuscript), or elaborate on this item by providing additional information not in the ms, or briefly explain why the item is not applicable/relevant for your study

Baseline demographic and clinical characteristics are described in the result section and with a table

15-i) Report demographics associated with digital divide issues

In ehealth trials it is particularly important to report demographics associated with digital divide issues, such as age, education, gender, social-economic status, computer/Internet/ehealth literacy of the participants, if known.

subitem not at all important

1 ○

2 ○

3 ○

4 ○

5 ◉

essential

Effacer la sélection

Does your paper address subitem 15-i? *

Copy and paste relevant sections from the manuscript (include quotes in quotation marks "like this" to indicate direct quotes from your manuscript), or elaborate on this item by providing additional information not in the ms, or briefly explain why the item is not applicable/relevant for your study

age, education, gender, social-economic status, computer/Internet/ehealth literacy of the participants are variables that are recorded in our questionnaires.

16) For each group, number of participants (denominator) included in each analysis and whether the analysis was by original assigned groups

16-i) Report multiple “denominators” and provide definitions

Report multiple “denominators” and provide definitions: Report N’s (and effect sizes) “across a range of study participation [and use] thresholds” [1], e.g., N exposed, N consented, N used more than x times, N used more than y weeks, N participants “used” the intervention/comparator at specific pre-defined time points of interest (in absolute and relative numbers per group). Always clearly define “use” of the intervention.

subitem not at all important

1 ○

2 ○

3 ○

4 ○

5 ◉

essential

Effacer la sélection

Does your paper address subitem 16-i? *

Copy and paste relevant sections from the manuscript (include quotes in quotation marks "like this" to indicate direct quotes from your manuscript), or elaborate on this item by providing additional information not in the ms, or briefly explain why the item is not applicable/relevant for your study

Data analysts of the Methods team took care of this

16-ii) Primary analysis should be intent-to-treat

Primary analysis should be intent-to-treat, secondary analyses could include comparing only “users”, with the appropriate caveats that this is no longer a randomized sample (see 18-i).

subitem not at all important

1 (selected)

2

3

4

5

essential

Effacer la sélection

Does your paper address subitem 16-ii?

Copy and paste relevant sections from the manuscript (include quotes in quotation marks "like this" to indicate direct quotes from your manuscript), or elaborate on this item by providing additional information not in the ms, or briefly explain why the item is not applicable/relevant for your study

Good idea to be considered for our next paper

17a) For each primary and secondary outcome, results for each group, and the estimated effect size and its precision (such as 95% confidence interval)

Does your paper address CONSORT subitem 17a? *

Copy and paste relevant sections from the manuscript (include quotes in quotation marks "like this" to indicate direct quotes from your manuscript), or elaborate on this item by providing additional information not in the ms, or briefly explain why the item is not applicable/relevant for your study

This has been precised in the paper !

17a-i) Presentation of process outcomes such as metrics of use and intensity of use

In addition to primary/secondary (clinical) outcomes, the presentation of process outcomes such as metrics of use and intensity of use (dose, exposure) and their operational definitions is critical. This does not only refer to metrics of attrition (13-b) (often a binary variable), but also to more continuous exposure metrics such as “average session length”. These must be accompanied by a technical description how a metric like a “session” is defined (e.g., timeout after idle time) [1] (report under item 6a).

subitem not at all important

- 1 (selected)
- 2
- 3
- 4
- 5

essential

Effacer la sélection

Does your paper address subitem 17a-i?

Copy and paste relevant sections from the manuscript (include quotes in quotation marks "like this" to indicate direct quotes from your manuscript), or elaborate on this item by providing additional information not in the ms, or briefly explain why the item is not applicable/relevant for your study

We were not into quantitative use but rather qualitative use and the latter is analysed by means of focus groups and interviews.

17b) For binary outcomes, presentation of both absolute and relative effect sizes is recommended

Does your paper address CONSORT subitem 17b? *

Copy and paste relevant sections from the manuscript (include quotes in quotation marks "like this" to indicate direct quotes from your manuscript), or elaborate on this item by providing additional information not in the ms, or briefly explain why the item is not applicable/relevant for your study

We do present absolute and relative effect sizes in our paper

18) Results of any other analyses performed, including subgroup analyses and adjusted analyses, distinguishing pre-specified from exploratory

Does your paper address CONSORT subitem 18? *

Copy and paste relevant sections from the manuscript (include quotes in quotation marks "like this" to indicate direct quotes from your manuscript), or elaborate on this item by providing additional information not in the ms, or briefly explain why the item is not applicable/relevant for your study

We do state when is the case subgroup analyses and adjusted analyses,

18-i) Subgroup analysis of comparing only users

A subgroup analysis of comparing only users is not uncommon in ehealth trials, but if done, it must be stressed that this is a self-selected sample and no longer an unbiased sample from a randomized trial (see 16-iii).

subitem not at all important

- 1 (selected)
- 2
- 3
- 4
- 5

essential

Effacer la sélection

Does your paper address subitem 18-i?

Copy and paste relevant sections from the manuscript (include quotes in quotation marks "like this" to indicate direct quotes from your manuscript), or elaborate on this item by providing additional information not in the ms, or briefly explain why the item is not applicable/relevant for your study

All participants were users. We describe biais in our paper

19) All important harms or unintended effects in each group

(for specific guidance see CONSORT for harms)

Does your paper address CONSORT subitem 19? *

Copy and paste relevant sections from the manuscript (include quotes in quotation marks "like this" to indicate direct quotes from your manuscript), or elaborate on this item by providing additional information not in the ms, or briefly explain why the item is not applicable/relevant for your study

No important harms or unintended effects were observed

19-i) Include privacy breaches, technical problems

Include privacy breaches, technical problems. This does not only include physical “harm” to participants, but also incidents such as perceived or real privacy breaches [1], technical problems, and other unexpected/unintended incidents. “Unintended effects” also includes unintended positive effects [2].

subitem not at all important

- 1 ○
- 2 ○
- 3 ◉
- 4 ○
- 5 ○

essential

Effacer la sélection

Does your paper address subitem 19-i?

Copy and paste relevant sections from the manuscript (include quotes in quotation marks "like this" to indicate direct quotes from your manuscript), or elaborate on this item by providing additional information not in the ms, or briefly explain why the item is not applicable/relevant for your study

Technical problems are mentionned. No privacy breaches were observed.

19-ii) Include qualitative feedback from participants or observations from staff/researchers

Include qualitative feedback from participants or observations from staff/researchers, if available, on strengths and shortcomings of the application, especially if they point to unintended/unexpected effects or uses. This includes (if available) reasons for why people did or did not use the application as intended by the developers.

subitem not at all important

1 ○

2 ○

3 ○

4 ○

5 ◉

essential

Effacer la sélection

Does your paper address subitem 19-ii?

Copy and paste relevant sections from the manuscript (include quotes in quotation marks "like this" to indicate direct quotes from your manuscript), or elaborate on this item by providing additional information not in the ms, or briefly explain why the item is not applicable/relevant for your study

We have 2 pages relating our qualitative analysis of our focus groups and interviews in which participants provide their feedback.

DISCUSSION

22) Interpretation consistent with results, balancing benefits and harms, and considering other relevant evidence

NPT: In addition, take into account the choice of the comparator, lack of or partial blinding, and unequal expertise of care providers or centers in each group

22-i) Restate study questions and summarize the answers suggested by the data, starting with primary outcomes and process outcomes (use)

Restate study questions and summarize the answers suggested by the data, starting with primary outcomes and process outcomes (use).

subitem not at all important

1 ○

2 ○

3 ○

4 ○

5 ◉

essential

Effacer la sélection

Does your paper address subitem 22-i? *

Copy and paste relevant sections from the manuscript (include quotes in quotation marks "like this" to indicate direct quotes from your manuscript), or elaborate on this item by providing additional information not in the ms, or briefly explain why the item is not applicable/relevant for your study

Yes, we have already done this.

22-ii) Highlight unanswered new questions, suggest future research

Highlight unanswered new questions, suggest future research.

subitem not at all important

1 ○

2 ○

3 ○

4 ◉

5 ○

essential

Effacer la sélection

Does your paper address subitem 22-ii?

Copy and paste relevant sections from the manuscript (include quotes in quotation marks "like this" to indicate direct quotes from your manuscript), or elaborate on this item by providing additional information not in the ms, or briefly explain why the item is not applicable/relevant for your study

This is done in the conclusion section.

20) Trial limitations, addressing sources of potential bias, imprecision, and, if relevant, multiplicity of analyses

20-i) Typical limitations in ehealth trials

Typical limitations in ehealth trials: Participants in ehealth trials are rarely blinded. Ehealth trials often look at a multiplicity of outcomes, increasing risk for a Type I error. Discuss biases due to non-use of the intervention/usability issues, biases through informed consent procedures, unexpected events.

subitem not at all important

1 ○

2 ○

3 ○

4 ○

5 ◉

essential

Effacer la sélection

Does your paper address subitem 20-i? *

Copy and paste relevant sections from the manuscript (include quotes in quotation marks "like this" to indicate direct quotes from your manuscript), or elaborate on this item by providing additional information not in the ms, or briefly explain why the item is not applicable/relevant for your study

In discussion section we reflect with honnestly on the potential bias and limitation of our RC trial

21) Generalisability (external validity, applicability) of the trial findings

NPT: External validity of the trial findings according to the intervention, comparators, patients, and care providers or centers involved in the trial

21-i) Generalizability to other populations

Generalizability to other populations: In particular, discuss generalizability to a general Internet population, outside of a RCT setting, and general patient population, including applicability of the study results for other organizations

subitem not at all important

1 ○

2 ○

3 ○

4 ○

5 ◉

essential

Effacer la sélection

Does your paper address subitem 21-i?

Copy and paste relevant sections from the manuscript (include quotes in quotation marks "like this" to indicate direct quotes from your manuscript), or elaborate on this item by providing additional information not in the ms, or briefly explain why the item is not applicable/relevant for your study

This is done in the discussion and conclusion sections.

21-ii) Discuss if there were elements in the RCT that would be different in a routine application setting

Discuss if there were elements in the RCT that would be different in a routine application setting (e.g., prompts/reminders, more human involvement, training sessions or other co-interventions) and what impact the omission of these elements could have on use, adoption, or outcomes if the intervention is applied outside of a RCT setting.

subitem not at all important

1 ○

2 ○

3 ○

4 ○

5 ◉

essential

Effacer la sélection

Does your paper address subitem 21-ii?

Copy and paste relevant sections from the manuscript (include quotes in quotation marks "like this" to indicate direct quotes from your manuscript), or elaborate on this item by providing additional information not in the ms, or briefly explain why the item is not applicable/relevant for your study

This is done in the discussion and conclusion sections

OTHER INFORMATION

23) Registration number and name of trial registry

Does your paper address CONSORT subitem 23? *

Copy and paste relevant sections from the manuscript (include quotes in quotation marks "like this" to indicate direct quotes from your manuscript), or elaborate on this item by providing additional information not in the ms, or briefly explain why the item is not applicable/relevant for your study

It is explicited in out paper

24) Where the full trial protocol can be accessed, if available

Does your paper address CONSORT subitem 24? *

Cite a Multimedia Appendix, other reference, or copy and paste relevant sections from the manuscript (include quotes in quotation marks "like this" to indicate direct quotes from your manuscript), or elaborate on this item by providing additional information not in the ms, or briefly explain why the item is not applicable/relevant for your study

YEs, it is described in the text, there is a diagram of it and we completed in the multimedia appendix

25) Sources of funding and other support (such as supply of drugs), role of funders

Does your paper address CONSORT subitem 25? *

Copy and paste relevant sections from the manuscript (include quotes in quotation marks "like this" to indicate direct quotes from your manuscript), or elaborate on this item by providing additional information not in the ms, or briefly explain why the item is not applicable/relevant for your study

We have explicited the funding and other support in our paper

X27) Conflicts of Interest (not a CONSORT item)

X27-i) State the relation of the study team towards the system being evaluated

In addition to the usual declaration of interests (financial or otherwise), also state the relation of the study team towards the system being evaluated, i.e., state if the authors/evaluators are distinct from or identical with the developers/sponsors of the intervention.

subitem not at all important

1 ○

2 ○

3 ○

4 ○

5 ◉

essential

Effacer la sélection

Does your paper address subitem X27-i?

Copy and paste relevant sections from the manuscript (include quotes in quotation marks "like this" to indicate direct quotes from your manuscript), or elaborate on this item by providing additional information not in the ms, or briefly explain why the item is not applicable/relevant for your study

This has been done

Your answer must have a minimum of 25 characters.

About the CONSORT EHEALTH checklist

As a result of using this checklist, did you make changes in your manuscript? *

- ○ yes, major changes
- ○ yes, minor changes
- ◉ no

What were the most important changes you made as a result of using this checklist?

We already had all the items of the checklist

How much time did you spend on going through the checklist INCLUDING making changes in your manuscript *

3 h going through this checklist!

As a result of using this checklist, do you think your manuscript has improved? *

- ○ yes
- ◉ no
- ○ Autre :

Would you like to become involved in the CONSORT EHEALTH group?

This would involve for example becoming involved in participating in a workshop and writing an "Explanation and Elaboration" document

- ( ) yes
- (•) no
- ( ) Autre :

Effacer la sélection

Any other comments or questions on CONSORT EHEALTH

Votre réponse

STOP - Save this form as PDF before you click submit

To generate a record that you filled in this form, we recommend to generate a PDF of this page (on a Mac, simply select "print" and then select "print as PDF") before you submit it.

When you submit your (revised) paper to JMIR, please upload the PDF as supplementary file.

Don't worry if some text in the textboxes is cut off, as we still have the complete information in our database. Thank you!

Final step: Click submit !

Click submit so we have your answers in our database!

Envoyer | Effacer le formulaire

N'envoyez jamais de mots de passe via Google Forms.

Ce contenu n'est ni rédigé, ni cautionné par Google. Signaler un cas d'utilisation abusive - Conditions d'utilisation - Règles de confidentialité

Google Forms
